# GULP1 Fuels Resistance to PD-1 Blockade through Lipid-Mediated Metabolic Reprogramming of Tumor-Associated Macrophages in Gastric Cancer

**DOI:** 10.34133/cancomm.0054

**Published:** 2026-10-06

**Authors:** Zihao Zhang, Wenxin Xu, Kuan Yu, Anran Bao, Yifan Cao, Mincheng Yu, Qiang Yu, Yufei Zhao, Liang Gong, Peiyi Xie, Zhenbin Shen, Jing Qin

**Affiliations:** ^1^ Department of General Surgery, Zhongshan Hospital, Fudan University, Shanghai, P. R. China.; ^2^ Gastric Cancer Center, Zhongshan Hospital, Fudan University, Shanghai, P. R. China.; ^3^ Department of Hepatobiliary Surgery and Transplantation, Liver Cancer Institute, Key Laboratory of Carcinogenesis and Cancer Invasion of Ministry of Education, Zhongshan Hospital, Fudan University, Shanghai, P. R. China.; ^4^ Department of Colorectal Surgery, Zhongshan Hospital, Fudan University, Shanghai, P. R. China.

## Abstract

**Background:** Immune checkpoint inhibitors targeting programmed cell death protein 1 (PD-1) have substantially improved the treatment of multiple cancers; however, only a small proportion of gastric cancer (GC) patients achieve durable clinical benefit. The underlying tumor-intrinsic mechanisms driving resistance to anti-PD-1 therapy remain poorly understood. This study aimed to identify key regulators of immunotherapy resistance in GC and to elucidate their functional and mechanistic roles. **Methods:** Transcriptomic data from anti-PD-1-responsive and anti-PD-1-resistant GC patients were analyzed and integrated with mouse model data and publicly available single-cell RNA sequencing datasets. Functional experiments, including in vitro assays and in vivo tumor models, were performed to validate candidate genes and explore their roles in tumor progression and immunotherapy resistance. **Results:** GULP PTB domain-containing engulfment adaptor 1 (GULP1) was identified as a key driver of resistance to anti-PD-1 therapy in GC. Elevated GULP1 expression was associated with poor prognosis and unfavorable clinical responses to PD-1 blockade. GULP1 promoted GC progression and attenuated the therapeutic efficacy of anti-PD-1 treatment. Functionally, GULP1 facilitated the accumulation and metabolic reprogramming of CD36^+^ programmed death-ligand 1 (PD-L1)^+^ tumor-associated macrophages (TAMs) by shaping a lipid-enriched tumor microenvironment, thereby impairing CD8^+^ T-cell function. Mechanistically, GULP1 promoted insulin-induced gene 2 (INSIG2) palmitoylation through the recruitment of zinc finger DHHC-type palmitoyltransferase 8 (zDHHC8), resulting in sterol regulatory element-binding protein 1 (SREBP1) nuclear translocation and enhanced de novo fatty acid biosynthesis. Importantly, pharmacological inhibition of Gulp1 with glycyrrhizic acid significantly restored sensitivity to anti-PD-1 therapy in vivo. **Conclusions:** Collectively, these findings uncovered a previously unrecognized GULP1–fatty acid–CD36^+^PD-L1^+^ TAM–CD8^+^ T-cell axis that drove resistance to PD-1 blockade in GC, highlighting GULP1 as a promising therapeutic target and predictive biomarker for immunotherapy responsiveness.

## Background

Gastric cancer (GC) remains a major global health challenge due to its high incidence and mortality, particularly across East and Southeast Asia [1]. As the fifth most frequently diagnosed malignancy worldwide and the fourth leading cause of cancer-related deaths, GC continues to impose a substantial disease burden despite advances in therapeutic interventions [2]. Immune checkpoint blockade (ICB), particularly targeting the programmed cell death protein 1 (PD-1) pathway, has revolutionized cancer treatment by reinvigorating cytotoxic CD8^+^ T cells and producing durable clinical responses across multiple tumor types [3–5]. Nevertheless, only a subset of GC patients derives meaningful benefit from PD-1 blockade [6]. Resistance—whether primary or acquired—arises from a constellation of tumor-intrinsic alterations, stromal and extracellular matrix constraints, and immunological mechanisms within the tumor microenvironment (TME) [7]. Although biomarkers such as microsatellite-instability-high status and Epstein–Barr virus positivity, which relate with higher tumor mutational burden, predict sensitivity to PD-1 inhibitors, the majority of GC cases remain refractory [8,9]. Therefore, defining molecular pathways that orchestrate immune evasion in GC remains an urgent unmet need. A deeper understanding of the molecular and cellular mechanisms underlying such resistance is therefore critical for improving the success of immunotherapy in GC.

Given the intricate interplay between tumor cells and the host immune system, immune evasion within the TME represents a central obstacle to effective immunotherapy [10]. Among the diverse immune components of the TME, tumor-associated macrophages (TAMs) play a decisive role in orchestrating tumor progression and modulating therapeutic responses [11–13]. TAMs display remarkable functional plasticity, adopting either tumor-promoting or tumor-suppressive phenotypes depending on environmental cues [14,15]. Increasing evidence suggests that macrophage metabolism plays a critical role in shaping their functional states. Immunosuppressive TAMs predominantly rely on lipid metabolism, whereas proinflammatory macrophages tend to favor glycolytic pathways [16,17]. However, the metabolic remodeling of TAMs in GC and its impact on immune checkpoint inhibitor resistance remain poorly characterized, hindering the rational design of metabolic or immunologic combination strategies.

GULP PTB domain-containing engulfment adaptor 1 (GULP1) is an adaptor protein implicated in various cellular processes and diseases, including several malignancies. Interestingly, the biological function of GULP1 appears to be context dependent. For example, in urothelial carcinoma, GULP1 suppresses tumor progression through inhibition of the nuclear factor erythroid 2-related factor 2 (NRF2)–Kelch-like ECH-associated protein 1 (KEAP1) axis [18], whereas in hepatocellular carcinoma, it promotes tumor growth by stabilizing β-catenin and activating epithelial–mesenchymal transition signaling [18,19]. Moreover, recent studies have associated GULP1 expression with immune cell infiltration and cytotoxic T-cell activity, suggesting its potential as a biomarker for immunotherapy responsiveness [20,21]. Despite these observations, the precise contribution of GULP1 to GC progression and its involvement in the immunotherapy resistance remain unexplored.

In the present study, we integrated analyses of anti-PD-1-resistant and anti-PD-1-sensitive GC patient samples, an anti-PD-1-resistant mouse model, and publicly available single-cell RNA sequencing (scRNA-seq) datasets to investigate the immunologic role of GULP1. In the present study, we aimed to investigate the tumor-intrinsic mechanisms underlying resistance to anti-PD-1 therapy in GC, with a particular focus on the potential immunologic role of GULP1. To this end, we integrated transcriptomic analyses of anti-PD-1-resistant and anti-PD-1-sensitive GC patient samples, an anti-PD-1-resistant mouse model, and publicly available scRNA-seq datasets to systematically evaluate the association between GULP1 and the TME.

## Materials and Methods

### Patient cohorts

The Zhongshan Gastric Cancer Immunotherapy (ZSGCI) cohort included 82 patients with unresectable GC who received anti-PD-1 based therapy at Zhongshan Hospital, Fudan University, between August 2022 and June 2024. Therapeutic efficacy was first evaluated by computed tomography (CT) imaging. Gastrectomy was performed in patients who met surgical criteria or experienced significant impairment in quality of life following anti-PD-1-based conversion therapy. The tumor specimen was evaluated by 2 pathologists according to tumor response grade (TRG) based on Japanese Gastric Cancer Association (JGCA) criteria [22], classifying responses as TRG3 (no residual tumor, 0%), TRG2 (1% to 33% residual tumor), TRG1b (33% to 67% residual tumor), TRG1a (>67% residual tumor), and TRG0 (no regression, 100% residual tumor). In the present study, patients with TRG2 and TRG3 were defined as responders, whereas those with TRG0, TRG1a, and TRG1b were defined as nonresponders. The clinicopathologic characteristics of patients in ZSGCI are summarized in Table S1. For transcriptomic profiling, GC tissues from 3 responders and 3 nonresponders were subjected to RNA sequencing (RNA-seq). An independent Zhongshan GC (ZSGC) cohort was included to assess protein expression and its association with patient prognosis. This cohort consisted of 135 patients with GC who underwent radical D2 gastrectomy between March 2010 and August 2012, with follow-up conducted until August 2022. The clinicopathologic characteristics of patients in the ZSGC cohort are summarized in Table S2. In addition, fresh tumor and adjacent normal tissues were obtained from 56 patients who underwent radical D2 gastrectomy between June 2023 and December 2024 for real-time quantitative reverse transcription polymerase chain reaction (RT-qPCR) and Western blotting assay [12]. All samples were collected with informed consent under protocols approved by the Ethics Committee of Zhongshan Hospital, Fudan University (No. B2024-261), and all procedures adhered to the principles of the Declaration of Helsinki.

### Cell lines and cell culture

The human GC cell lines AGS, MKN-45, MKN-28, HGC-27, and human gastric epithelial cell line GES-1 were provided by the Gastric Cancer Center, Zhongshan Hospital, Fudan University. All cell lines used in this study were obtained from authenticated cell banks. According to the information provided by the cell banks, the cell lines had been authenticated by short tandem repeat profiling. The murine GC cell line YTN-16 was generously provided by Prof. Sachiyo Nomura (University of Tokyo, Japan). YTN-16 cells were cultured in Dulbecco’s modified Eagle’s medium (DMEM; C11965500BT, Gibco, Thermo Fisher Scientific), while the human GC cell lines and GES-1 were maintained in RPMI 1640 medium (C11875500BT, Gibco). All cell cultures were supplemented with 10% fetal bovine serum (10099141C, Gibco) and 100 U/ml penicillin–streptomycin (C0223, Beyotime). Cells were incubated at 37 °C in a humidified atmosphere containing 5% CO_2_. For the generation of bone-marrow-derived macrophages (BMDMs), bone marrow cells were flushed from the femurs and tibias of 6- to 8-week-old C57BL/6 mice and cultured in RPMI 1640 medium supplemented with 10 ng/ml macrophage colony-stimulating factor (576408, BioLegend) for 7 d to induce macrophage differentiation. The expression of CD11b and F4/80 was analyzed by flow cytometry, and macrophage preparations with a purity exceeding 95% were used for downstream experiments. For co-culture assays, BMDMs were exposed to conditioned media derived from GC cells for 72 h using transwell plates. Where indicated, etomoxir (T4535L, 200 μmol/l, Topscience) or glycyrrhizic acid (GA, HY-N0184; 10 μmol/l, MedChemExpress) was added during co-culture. After incubation, macrophages were collected for further functional and molecular analyses.

### Lentivirus transduction and cell line construction

To generate *GULP1* (*Gulp1*)-overexpressing, *Gulp1*-knockout, *GULP1*-knockdown, and corresponding control tumor cell lines, GC cells were infected with lentiviral particles carrying the *GULP1* (*Gulp1*) overexpression construct, single guide RNA targeting *Gulp1*, short hairpin RNA targeting *GULP1*, or the corresponding empty vector/control constructs. After infection, cells were selected with puromycin (2 μg/ml) for 14 d to obtain stable cell populations. Stable cells were maintained under puromycin selection and used for subsequent experiments. The efficiency of GULP1 overexpression, knockout, or knockdown was confirmed by RT-qPCR and/or Western blotting analysis.

### Animal experiment

Male C57BL/6 and BALB/c nude mice aged 5 to 6 weeks were obtained from the Shanghai Model Organisms Center. The myeloid-specific *Cd36*-knockout mice were generated by crossing *Lyz2*-Cre mice with *Cd36*^fl/fl^ mice purchased from the Shanghai Model Organisms Center. Genotyping for *Lyz2*-Cre and *Cd36*^fl/fl^ alleles was conducted using conventional PCR assays, and the primer sequences are listed in Table S3. All strains were maintained on a C57BL/6 genetic background, and littermate or cage-matched animals were used as experimental controls. Mice were housed under specific-pathogen-free conditions with ad libitum access to food and water. For subcutaneous tumor xenografts, AGS or YTN-16 cells (5 × 10^6^) were injected into the right inguinal region of BALB/c nude or C57BL/6 mice. Tumor volumes were calculated according to the formula

$$\text{Volume}=0.5\times \text{length}\times {\left(\text{width}\right)}^{2}$$ (1)

For the orthotopic GC model, YTN-16 cells (5 × 10^5^) were inoculated into the subserosal layer of the stomach in C57BL/6 mice. An orthotopic gastric tumor model for survival analysis was established by coinjecting YTN-16 cells with primary mouse fibroblasts at a ratio of 7:3 into the gastric serosal layer. This coinjection strategy was applied to facilitate tumor establishment in vivo. Tumor progression was monitored using in vivo bioluminescence imaging (IVIS Spectrum, PerkinElmer). For antibody-based therapy experiments, mice received intraperitoneal injections of either immunoglobulin G2a (IgG2a) isotype control (100 μg/mouse, BE0089, Bio X Cell) or anti-PD-1 antibody (200 μg/mouse, BE0146, Bio X Cell) 3 times weekly for 3 consecutive weeks. GA (50 mg/kg) was administered intraperitoneally once daily. Humane endpoints were defined based on institutional guidelines, including significant weight loss (>20%), impaired mobility, ulceration, or tumor burden exceeding ethical limits. Mice were closely monitored throughout the experiments, and animals reaching these criteria were humanely euthanized. Euthanasia was performed by CO_2_ inhalation followed by cervical dislocation to ensure death, in accordance with institutional animal care and use guidelines. Tumor tissues were harvested for subsequent analyses. All animal procedures complied with the Animal Research: Reporting of *In Vivo* Experiments guidelines and were approved by the Institutional Animal Care and Ethics Committee of Zhongshan Hospital, Fudan University (Y2023-190).

### Patient-derived organoid model

Patient-derived organoid (PDO) models were established from GC specimens collected after surgical resection at Zhongshan Hospital, Fudan University. Fresh tumor tissues were promptly transferred on ice and mechanically dissociated into small fragments in 10-cm culture dishes using sterile scalpels and forceps. The minced tissues were then suspended in DMEM supplemented with 10% fetal bovine serum, 100 U/ml collagenase type IV (C5138, Sigma-Aldrich), and 125 μg/ml Dispase II (D4693, Sigma-Aldrich). The digestion was performed at 37 °C for 30 min on a rotating platform. Following enzymatic digestion, red blood cells were removed using RBC Lysis Buffer (C3702, Beyotime). The digested suspension was filtered through 100- and 40-μm cell strainers to collect the 40- to 100-μm (S2) fractions. After centrifugation at 300×*g* at room temperature for 3 min, supernatants containing debris and residual enzymes were discarded, and the cell pellets were reserved for organoid culture. The S2 fraction was resuspended in type I rat tail collagen at a final concentration of 2.5 mg/ml, prepared using 10× phosphate-buffered saline (PBS) containing phenol red. The pH was adjusted to 7.0 to 7.5 with NaOH and verified using PANPEHA Whatman indicator paper. The spheroid–collagen mixture was gently loaded into the central gel chamber of a 3-dimensional (3D) microfluidic culture device to form the PDO matrix. For pharmacological treatments, PDOs were incubated with anti-PD-1 antibody (250 μg/ml, GA (50 μmol/l), and isotype-matched human IgG2a antibody (250 μg/ml). Peripheral blood mononuclear cells isolated from the same patients were subjected to magnetic sorting of monocytes and CD8^+^ T cells using anti-CD14 microbeads and anti-CD8 microbeads (17853, STEMCELL Technologies). CD8^+^ T cells and macrophages were obtained from matched patient samples at distinct time points. PDOs were co-cultured with macrophages at a tumor cell-to-macrophage ratio of 2:1. After 24 h of co-culture, T cells were added at a T cell-to-tumor cell ratio of 5:1, and the triculture system was maintained for an additional 72 h. For treatment conditions, anti-PD-1 antibody (250 μg/ml) and GA (50 μmol/l) were added at the initiation of the T-cell co-culture phase. Where indicated, treatments were replenished during medium replacement. All experimental conditions were established based on previously reported organoid-immune co-culture protocols [23].

### Cytometry by time of flight

Cytometry by time of flight (CyTOF) sample preparation, staining, acquisition, and data analysis were performed as follows: tumor tissues were minced into ~1-mm^3^ fragments and enzymatically digested by the Tumor Tissue Dissociation Kit (abs50090-10T, Absin) at 37 °C for 1 h, followed by filtration through a 70-μm cell strainer to obtain single-cell suspensions. Cells were washed with Cell Staining Buffer, centrifuged at 300×*g* for 5 min at 2 to 8 °C, and counted. For CyTOF staining, cells were first labeled with ^194^Pt for viability discrimination, followed by Fc receptor blocking. Surface markers were stained using antibody cocktails for 30 min, after which cells were fixed, permeabilized, and stained with intracellular antibodies. Cells were then incubated with a DNA intercalator overnight for nucleic acid labeling and doublet discrimination. Sample barcoding was performed using metal isotope barcodes according to the manufacturer’s instructions, and cells were resuspended in deionized water for acquisition. Prior to acquisition, the CyTOF instrument (Helios3 mass cytometer, PLTTech) was calibrated using standard tuning and quality control procedures, and EQ Four Element Calibration Beads were added for normalization. Raw data were debarcoded using a doublet-filtering scheme and normalized across batches using bead-based normalization. Cells were manually gated using the FlowJo software (v10.8.1, BD Biosciences, Ashland, OR, USA; https://www.flowjo.com) to exclude debris, dead cells, and doublets, retaining live single immune cells for downstream analysis. High-dimensional data were analyzed using the X-shift clustering algorithm to identify distinct cell populations, which were annotated based on canonical marker expression patterns. Dimensionality reduction was performed using t-distributed stochastic neighbor embedding for visualization, and statistical comparisons of cell population frequencies between groups were conducted using the Student *t* test. A comprehensive list of the antibodies used is provided in Table S4.

### Flow cytometry

Fresh tumor specimens from mouse models were finely minced and enzymatically dissociated into single-cell suspensions using the Tumor Dissociation Kit, following the manufacturer’s protocol. Cultured cells were detached with trypsin and subsequently resuspended in PBS. After labeling with a fixable viability dye (423101, Zombie Aqua Fixable Viability Kit, BioLegend), the cells were fixed and permeabilized using the Fixation and Permeabilization Solution (554722, BD Biosciences). Cells were then incubated with fluorochrome-conjugated antibodies for 30 min on ice in the dark. Flow cytometric data were acquired on a BD LSRFortessa X-20 cytometer and analyzed using the FlowJo software. Details of the antibodies used are provided in Table S5.

### Acyl–biotin exchange assay

Cells were lysed in a buffer consisting of 50 mmol/l Tris–HCl (pH 7.5), 150 mmol/l NaCl, 1 mmol/l MgCl_2_, 1% NP-40, and protease inhibitors. The lysates were incubated with 50 mmol/l *N*-ethylmaleimide at 4 °C for 1.5 h with gentle rotation to block free thiols. Samples were then mixed with the appropriate primary antibody and magnetic beads and allowed to bind for 3 h at 4 °C. Bead–protein complexes were rinsed 5 times with the initial lysis buffer and subsequently washed twice with a slightly modified buffer (50 mmol/l Tris–HCl, pH 7.2; 150 mmol/l NaCl; and 1 mmol/l MgCl_2_). To cleave thioester bonds, the beads were incubated for 1 h in buffer (50 mmol/l Tris–HCl, pH 7.2; 150 mmol/l NaCl; 1 mmol/l MgCl_2_; 10% glycerol; and protease inhibitors) supplemented with 1 mol/l hydroxylamine (HAM). Afterward, the beads were washed 4 times with the same pH 7.2 buffer and an additional 3 times with a buffer adjusted to pH 6.2 (50 mmol/l Tris–HCl, 150 mmol/l NaCl, and 1 mmol/l MgCl_2_). For biotin labeling of newly exposed cysteines, bead-bound proteins were incubated with 2 μmol/l biotin-1,3-maleimidomethyl cyclohexanecarboxylate in the pH 6.2 buffer for 1 h at 4 °C. Finally, the beads were washed 3 times, and the eluted proteins were analyzed by Western blotting.

### Multiplex immunofluorescence staining

Tumor tissues were fixed in 4% paraformaldehyde and embedded in paraffin. Sections of 3-μm thickness were deparaffinized and rehydrated through graded ethanol series and xylene washes prior to multiplex immunofluorescence (mIF) staining. Antigen retrieval was carried out by microwave heating, followed by treatment with 3% hydrogen peroxide and blocking of nonspecific binding sites. The sections were then incubated with the corresponding primary antibodies, followed by horseradish peroxidase (HRP)-conjugated secondary antibodies. Fluorescent signals were visualized using an mIF staining kit, and nuclei were counterstained with 4′,6-diamidino-2-phenylindole (DAPI; P0131, Beyotime). Images were acquired using the Pannoramic MIDI II scanner (3DHISTECH, Hungary). Detailed information about the primary antibodies and the multiplex staining kit is provided in Table S5. Cells were fixed and blocked using the indicated reagents (P0098 and P0260, Beyotime). Subsequently, the cells were incubated with the corresponding primary antibodies overnight at 4 °C. After washing, fluorophore-conjugated secondary antibodies were added and incubated for 1 h at room temperature. Nuclei were counterstained with DAPI for 15 min. Fluorescent images were acquired using a confocal microscope and analyzed with the ImageJ software (v1.54t, National Institutes of Health, Bethesda, MD, USA; https://imagej.net/ij/).

### Immunohistochemistry

Tissue sections were deparaffinized, rehydrated, and subjected to antigen retrieval using ethylenediaminetetraacetic acid (P0085, Beyotime) buffer. After blocking with 5% bovine serum albumin (ST023, Beyotime) for 30 min, the slides were sequentially incubated with primary antibodies followed by an HRP-conjugated secondary antibody. Protein expression was quantified using the H-score, calculated as ∑(*P_i_* × *i*) = (percentage of weakly stained cells × 1) + (percentage of moderately stained cells × 2) + (percentage of strongly stained cells × 3), where *i* indicates staining intensity: negative (no staining) = 0, weak (light yellow) = 1, moderate (brownish yellow) = 2, and strong (dark brown) = 3, and *P_i_* represents the proportion of cells at each intensity. Cases with H-scores above the cohort median were classified as high expression, while those below the median were considered low expression. H-scores were independently assessed by 2 pathologists blinded to the patients’ clinical information. Detailed information on the antibodies used is provided in Table S5.

### Western blotting

Western blotting is performed as previously reported [24]. Detailed information about the primary antibodies is provided in Table S5.

### Co-immunoprecipitation

Co-immunoprecipitation (Co-IP) assays were performed using a Protein A/G immunoprecipitation kit (YJ201, Epizyme Biotech) according to the manufacturer’s instructions.

### Co-culture assay

A sterile transwell insert equipped with a 0.4-μm-pore polycarbonate membrane (3413, Corning) was employed to establish the in vitro co-culture system. Briefly, BMDMs (5 × 10^5^ cells) isolated from C57BL/6 mice were seeded into the lower chamber, while GC cells were plated in the upper compartment. After the indicated co-culture period, both the cells and culture supernatants were collected for subsequent analyses.

### RNA extraction and quantitative real-time PCR

FastPure Cell/Tissue Total RNA Isolation Kit V2, HiScript III All-in-One RT SuperMix Perfect for qPCR, and ChamQ Universal SYBR qPCR Master Mix (RC112-01, R333-01, and Q311-02, Vazyme Biotech Co., Ltd., Nanjing, China) were used as previously reported [12]. The expression of glyceraldehyde-3-phosphate dehydrogenase was used as an internal reference. The primers sequences used for the qPCR are provided in Table S3.

### Enzyme-linked immunosorbent assay

The culture supernatants of CD8^+^ T cells were harvested after centrifugation at 300×*g*, 5 min at room temperature, and cytokine levels were quantified using the IFN gamma ELISA Kit (A41150, Thermo Fisher Scientific) following the manufacturer’s protocols.

### Cellular energy metabolism analysis

For assessment of fatty acid uptake, cells were incubated in RPMI 1640 medium supplemented with 0.5 mmol/l BODIPY FL C12 (D3822, Thermo Fisher Scientific) at 37 °C in a 5% CO_2_ atmosphere for 20 min. Following incubation, cells were rinsed and resuspended in PBS for subsequent surface marker staining. To evaluate intracellular lipid accumulation, macrophages were stained with BODIPY 493/503 (D3922, Thermo Fisher Scientific) at a final concentration of 500 ng/ml for 20 min at 4 °C in the dark; after washing, nuclei were counterstained with DAPI, and images were captured using a confocal microscope. Mitochondrial respiration was assessed by measuring the oxygen consumption rate (OCR) using Seahorse XFp Extracellular Flux Analyzer (Agilent Seahorse Bioscience). Briefly, cells were cultured under the indicated conditions and seeded into Cell-Tak-coated Seahorse microplates in Seahorse XF Base Medium DMEM without phenol red, supplemented with 1 mmol/l sodium pyruvate, 2 mmol/l glutamine, and 10 mmol/l glucose. Before cell seeding, plates were precoated with Cell-Tak according to the manufacturer’s instructions. After plating, cells were incubated at 37 °C in a non-CO_2_ incubator for 30 min to allow attachment, followed by the addition of assay medium and further equilibration in a CO_2_-free incubator. Where indicated, metabolic inhibitors were added before OCR measurement. Mitochondrial respiration was analyzed by sequential injection of oligomycin, carbonyl cyanide-p-trifluoromethoxyphenylhydrazone (FCCP), and rotenone/antimycin A. OCR values were recorded over time and analyzed using the Seahorse Wave software.

### T-cell suppression assay

Naïve CD3^+^ T cells were isolated from the spleens of C57BL/6 mice using the EasySep Mouse T Cell Isolation Kit (STEMCELL Technologies). Purified T cells were then labeled with 5 mmol/l carboxyfluorescein succinimidyl ester (CFSE; 65-0850-84, Thermo Fisher Scientific) and preactivated for 24 h in plates coated with anti-CD3 (1 μg/ml, 553057, BD) and anti-CD28 (1 μg/ml, 553294, BD) antibodies at 37 °C under 5% CO_2_. Macrophages used for co-culture were derived from several sources:

• BMDMs obtained from naïve mice and co-cultured for 48 h with GULP1-overexpressing or vector tumor cells
• TAMs isolated from *Cd36*^fl/fl^*Lyz2*^Cre^ and *Cd36*^fl/fl^ mice, subsequently co-cultured with GULP1-overexpressing or vector control tumor cells for 48 h

Following these preconditioning steps, macrophages were co-cultured with preactivated splenic T cells at a 1:1 ratio in RPMI 1640 medium for 72 h at 37 °C in a 5% CO_2_ incubator. T-cell proliferation was evaluated by flow cytometry based on CFSE dilution within viable CD45^+^CD3^+^ T-cell populations.

### Structure-based virtual screening

To identify potential small-molecule candidates, a large-scale virtual screening was performed using MCE Bioactive Compound Library Plus (HY-L001P, MCE) comprising approximately 7.53 million compounds. Grid-based molecular docking was executed with the GLIDE module implemented in Schrödinger Maestro (version 11.4), focusing precisely on the predicted binding pockets. The 40 top-ranking compounds, as determined by docking scores, were subsequently obtained from MedChemExpress for further experimental validation.

### Surface plasmon resonance assays

Purified GULP1 protein was diluted in 10 mmol/l sodium acetate buffer (pH 4.5) to a final concentration of 200 μg/ml. The protein was then covalently immobilized onto a CM5 sensor chip using an amine-coupling kit (BR100050, Cytiva), reaching approximately 10,000 response units per flow cell. Binding analyses were performed on a Biacore 8K system (Cytiva) at 25 °C, with 4-(2-hydroxyethyl)-1-piperazineethane-sulfonic acid (Hepes)-buffered saline (20 mmol/l Hepes, pH 7.5, 200 mmol/l NaCl) supplemented with 1% dimethyl sulfoxide (ST038, Beyotime) as the running buffer. A series of increasing concentrations of test compounds were injected over the chip surface for 180 s at a flow rate of 30 μl/min, followed by a 330-s dissociation phase. The resulting sensorgrams were processed and fitted using the Biacore Insight Evaluation software, and equilibrium dissociation constants (*K*_D_) were determined based on a 1:1 steady-state binding model.

### Bulk RNA-seq and data analysis

Total RNA was isolated from murine tumor tissues or cells derived from mice using TRIzol reagent (Thermo Fisher Scientific). Subsequently, 2 μg of RNA from each sample was utilized for the construction of strand-specific RNA-seq libraries, which were prepared and sequenced by SeqHealth Co., Ltd. Raw reads were quality-trimmed using Trimmomatic (v0.36) and aligned to the mouse reference genome with STAR (v2.5.3a). The TCGA-Stomach Adenocarcinoma (STAD) dataset used in this study was obtained from The Cancer Genome Atlas (TCGA) via the Genomic Data Commons Data Portal (https://portal.gdc.cancer.gov/projects/TCGA-STAD) and was used for analysis. A publicly available cohort of unresectable GC patients treated with anti-PD-1 monotherapy from Samsung Medical Center–Sungkyunkwan University (SKKU-SMC cohort; PRJEB25780) [25] was used to independently evaluate the clinical predictive value of GULP1. Differential gene expression analysis between experimental groups was performed using the edgeR package (v3.12.1). Genes exhibiting an adjusted *P* value <0.05 and an absolute fold change ≥2 were considered significantly differentially expressed. Gene set enrichment analysis (GSEA) was carried out with the Broad Institute’s GSEA software. Functional enrichment of differentially expressed genes (DEGs), including Gene Ontology and Kyoto Encyclopedia of Genes and Genomes (KEGG) pathway analyses, was conducted using KOBAS (v2.1.1). Receiver operating characteristic (ROC) curve analysis was performed to evaluate the predictive performance of the variables individually and in combination. The area under the ROC curve (AUC) was calculated to quantify the discriminatory ability of each model. Calibration analysis and decision curve analysis were subsequently conducted to assess the predictive accuracy and potential clinical utility of the combined model.

### scRNA-seq data processing

scRNA-seq datasets (GSE206785 and GSE183904) were analyzed using the Seurat package (v4.0) in R. The analytical workflow included data quality control, normalization, identification of variable genes, principal component analysis, cell clustering, and dimensionality reduction via uniform manifold approximation and projection (UMAP). All analyses were performed with default parameters unless otherwise indicated. Low-quality cells were filtered out according to the following criteria: fewer than 300 or more than 3,000 detected genes per cell, fewer than 3 cells per gene, or mitochondrial gene content exceeding 10%. For cell type identification, highly variable genes were selected and subjected to principal component analysis. Statistically significant principal components were determined by JackStraw analysis and heatmap visualization, with principal components 1 to 20 retained for subsequent UMAP projection. Graph-based clustering was conducted using principal components 1 to 30 at a resolution of 0.2 to delineate distinct cellular populations. Visualization of gene expression patterns was performed using the Seurat functions VlnPlot, FeaturePlot, DotPlot, and DoHeatmap, which generate violin plots, feature plots, dot plots, and heatmaps, respectively. DEGs among clusters were identified using the FindAllMarkers function in Seurat, with the following parameters: min.pct = 0.25, logfc.threshold = 0.25, and only.pos = TRUE. The nonparametric Wilcoxon rank-sum test was applied to calculate *P* values, and multiple testing correction was performed using the Bonferroni method. Scaled and log-transformed expression values were visualized in heatmaps to display DEGs across clusters. Intercellular communication networks among distinct cell populations were inferred using CellChat [26]. This computational framework enables quantitative analysis of cell–cell interactions based on scRNA-seq data. Using this approach, we characterized both incoming signaling patterns in target cells and outgoing signaling activities from source cell populations. Ligand–receptor interactions with a *P* value <0.05 were considered statistically significant across different cell types. scRNA-seq expression matrices and corresponding cell cluster annotations were obtained from publicly available datasets. Gene sets representing specific metabolic processes were retrieved from the MSigDB database. A TME-metabolism-related algorithm was built based on the methods provided by Zhu et al. [27]. The AUCell algorithm was applied to quantify pathway activity at the single-cell level based on the expression of genes within each gene set, and cells were subsequently stratified into high- and low-activity groups according to their enrichment scores. The distribution of canonical marker genes across different cell populations was visualized using the DotPlot function in the Seurat package, and differential expression analysis was performed to identify cluster-specific markers. To evaluate metabolism-associated cell–cell interactions, pathway activity scores were aggregated at the cell cluster level. For each pair of cell clusters, an interaction strength score was calculated as the product of the summed activity scores of the sender and receiver clusters. Statistical significance was assessed using a permutation-based approach, in which cell cluster labels were randomly shuffled 1,000 times to generate a null distribution of interaction scores. The empirical *P* values were then calculated by comparing the observed interaction strength with the null distribution.

### Liquid chromatography–tandem mass spectrometry

For proteomic analysis, protein samples were extracted and quantified, followed by enzymatic digestion into peptides using trypsin according to standard procedures. The resulting peptides were separated by liquid chromatography–tandem mass spectrometry (LC–MS/MS). MS/MS data were acquired in a data-dependent acquisition mode, and raw files were processed against the corresponding species protein database using appropriate search software for peptide and protein identification. Only proteins identified with standard confidence criteria were retained for downstream analysis. Relative protein abundance was quantified based on normalized peptide signal intensity, and differentially expressed proteins were determined according to the predefined fold-change and statistical thresholds.

### Gas chromatography–mass spectrometry

Metabolites were extracted from cell samples using 1 ml of chloroform/methanol (2:1, v/v) and homogenized with glass beads. Following centrifugation at 12,000×*g* for 10 min at 4 °C, the supernatants were collected and subjected to derivatization by adding 2 ml of 1% sulfuric acid in methanol and 1 ml of *n*-hexane, followed by incubation at 80 °C for 30 min. After cooling, samples were washed with 5 ml of H_2_O and centrifuged at 3,000×*g* for 5 min at room temperature. The upper organic phase (700 μl) was transferred to a new tube and dried using 100 mg of anhydrous sodium sulfate. The resulting extracts were spiked with 15 μl of methyl salicylate (500 ppm) as an internal standard and subjected to gas chromatography–mass spectrometry (GC–MS) analysis. GC–MS analysis was performed using a Trace 1300 gas chromatograph coupled with an ISQ 7000 mass spectrometer (Thermo Fisher Scientific, USA). Separation was achieved on a Thermo TG-FAME capillary column, with helium as the carrier gas at a flow rate of 0.63 ml/min. Samples (1 μl) were injected in split mode (8:1). Mass spectrometric detection was conducted in electron impact ionization mode at 70 eV, and metabolites were analyzed using single ion monitoring mode.

### Statistical analyses

Quantitative data are presented as mean ± standard deviation. Statistical analyses were performed using GraphPad Prism (v9.0, GraphPad Software). Differences between 2 groups were evaluated using unpaired 2-tailed Student *t* tests, while comparisons among multiple groups were assessed using 1-way or 2-way analysis of variance followed by appropriate multiple comparison corrections. For categorical data, including the comparison of immunohistochemistry staining intensity between tumor and adjacent normal tissues, the chi-square (χ^2^) test was applied. Correlations between variables were analyzed using Spearman’s rank correlation. For in vivo experiments, mice were randomly assigned to treatment groups after tumor establishment to minimize selection bias. The number of animals in each group (*n*) is indicated in the corresponding figure legends. Investigators were not formally blinded to group allocation during treatment administration; however, tumor measurements and survival endpoints were assessed using predefined criteria to reduce potential bias. Overall survival (OS) and progression-free interval (PFI) were analyzed using the Kaplan–Meier method, and statistical significance was determined by the log-rank test. OS was defined as the time from diagnosis to death from any cause or last follow-up, and PFI was defined as the interval from diagnosis to the first occurrence of a progression-related event, including disease progression, recurrence, distant metastasis, new primary tumor, or death with tumor. Patients without an event at the last follow-up were censored. A 2-sided *P* value <0.05 was considered statistically significant.

## Results

### GULP1 was overexpressed in anti-PD-1-resistant GC patients and associated with a poor prognosis

To elucidate the genomic features of GC patients receiving neoadjuvant anti-PD-1 therapy, we first performed RNA-seq on tumor specimens collected from 6 GC patients (3 responders and 3 nonresponders) treated at Zhongshan Hospital. Treatment efficacy was first monitored by CT imaging (Fig. 1A) and confirmed based on TRG according to the JGCA criteria (Fig. 1B). The volcano plot shows the DEGs between responders and nonresponders (Fig. S1A), and single-sample GSEA (ssGSEA) revealed that responders harbored a more immunostimulatory TME compared with nonresponders (Fig. 1C). To establish an orthotopic GC mouse model with resistance to anti-PD-1 therapy and to explore the underlying molecular mechanisms, orthotopic GC mice were subjected to prolonged anti-PD-1 antibody treatment (Fig. 1D). Although tumor growth was largely suppressed, 3 tumors exhibited progressive growth despite treatment (Fig. 1E). Tumor tissues that were either sensitive or resistant to anti-PD-1 therapy were harvested at week 14 for RNA-seq, and the volcano plot shows the DEGs between anti-PD-1-resistant and anti-PD-1-sensitive tumors (Fig. S1B). To gain deeper insights into tumor-intrinsic genetic alterations with anti-PD-1 resistance, we analyzed publicly available scRNA-seq data from the GSE206785 dataset. Dimensionality reduction and clustering identified 9 distinct cell populations based on canonical marker genes (Fig. 1F and Fig. S1C). A total of 98 marker genes for malignant cells with prognostic values were selected for further screening (Fig. S1D). By integrating the up-regulated genes from anti-PD-1 nonresponder GC patients and resistant mouse tumors with these prognostic markers of malignant cells, we identified a set of overlapping candidate genes potentially associated with tumor-intrinsic resistance (Fig. S1E). Among these candidates, GULP1 exhibited the highest level of up-regulation in nonresponder patients (Fig. S1F and G). Moreover, analysis of the TCGA-STAD clinical dataset showed that elevated *GULP1* expression was significantly associated with poorer OS and PFI in GC patients (Fig. 1G), suggesting its strong clinical relevance. Therefore, based on its marked enrichment in anti-PD-1 nonresponders, its consistent association with unfavorable patient prognosis, and its potential role as a tumor-intrinsic resistance-associated gene, GULP1 was selected as the primary candidate for subsequent functional and mechanistic studies. Given that the efficacy of cancer immunotherapy largely depends on CD8^+^ T-cell infiltration within the TME, we next evaluated GULP1 expression in GC tissues and its correlation with CD8^+^ T-cell density. In the ZSGC cohort, immunohistochemistry revealed that GULP1 was predominantly expressed in tumor tissues compared with adjacent normal tissues (Fig. 1H and I), which was further confirmed by qPCR and Western blotting in fresh tumor and adjacent normal tissues obtained from 56 GC patients who underwent radical D2 gastrectomy at Zhongshan Hospital, Fudan University (Fig. S1H and I); besides that, its higher expression was inversely correlated with CD8^+^ T-cell infiltration (Fig. 1H and I). Accordingly, survival analysis showed that elevated GULP1 expression was significantly associated with worse OS in GC patients (Fig. 1J). To further validate these observations, we analyzed the ZSGCI cohort. The proportion of GULP1-high patients was higher in nonresponders (Fig. 1B). In addition, the intensity of GULP1 expression was markedly increased, whereas CD8^+^ T-cell infiltration was substantially reduced in nonresponders (Fig. 1K). Moreover, pretreatment samples demonstrated that nonresponders expressed higher levels of GULP1 than responders before treatment (Fig. S1J), with GULP1 further up-regulated following anti-PD-1 based therapy in nonresponders, while responders displayed a decreasing trend (*P* > 0.05) (Fig. 1L and M). To further evaluate the clinical predictive value of GULP1, we analyzed the SKKU-SMC cohort. ROC/AUC analysis showed that GULP1 displayed a predictive value for anti-PD-1 response and outperformed several established biomarkers, including microsatellite instability and Epstein–Barr virus status (Fig. S1K). Notably, combining GULP1 with PD-L1 expression further improved the predictive performance, suggesting that GULP1 may complement existing biomarkers for identifying GC patients more likely to benefit from PD-1 blockade. Together, these findings suggest that GULP1 functions as an adverse prognostic indicator in GC, associated with reduced CD8^+^ T-cell infiltration and a diminished response to anti-PD-1 therapy.

**Fig. 1.**
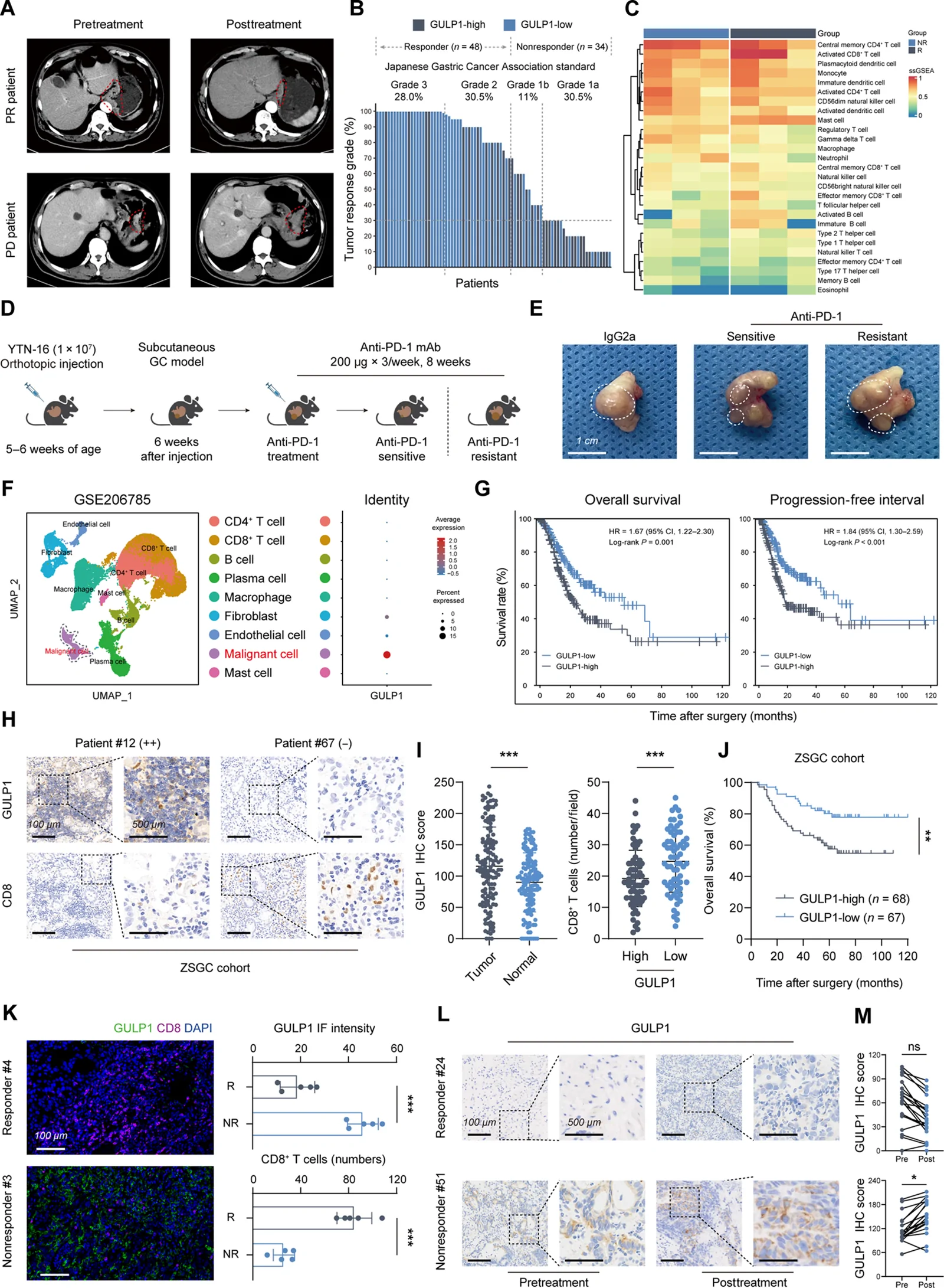
GULP1 is related to therapeutic efficacy in GC patients receiving anti-PD-1 based therapy. (A) Representative CT scans of the target lesions in partial response and progression disease GC patient; tumor lesions are indicated by red dashed lines. (B) Tumor regression level of specimen from GC patients in the ZSGCI cohort and corresponding gene expression level. (C) Heatmap of ssGSEA scores related to immune response between responder and nonresponder GC patients receiving anti-PD-1 based immunotherapy. (D and E) Schematic of establishing anti-PD-1 resistance mouse model and representative images of stomach tissues from mice in the indicated groups (*n* = 12). White dashed lines delineate the orthotopic gastric tumor regions. (F) UMAP plot of all cell types from GSE206785 and GULP1 expression level in different cell types. (G) Overall survival and progression-free interval of GC patients in the TCGA-STAD cohort according to GULP1 expression. (H and I) Representative IHC image and quantification of the GULP1 and CD8 expression levels of GC patients in the ZSGC cohort. (J) Survival analysis of patients in ZSGC according to GULP1 expression. (K) Representative mIF image and quantification of GULP1 expression and CD8^+^ T cells of responders and nonresponders from ZSGCI cohort. (L and M) Representative image and quantification of GULP1 of paired pre- and posttreatment specimens from responders and nonresponders in the ZSGCI cohort. **P < 0.01; ***P < 0.001; ns, not significant. Abbreviations: CT, computed tomography; DAPI, 4′,6-diamidino-2-phenylindole; GC, gastric cancer; GSE, Gene Expression Omnibus series; GULP1, GULP PTB domain-containing engulfment adaptor 1; HR, hazard ratio; IHC, immunohistochemistry; mIF, multiplex immunofluorescence; PD, progressive disease; PD-1, programmed cell death protein 1; PR, partial response; ssGSEA, single-sample gene set enrichment analysis; TCGA, The Cancer Genome Atlas; STAD, Stomach Adenocarcinoma; UMAP, uniform manifold approximation and projection; ZSGC, Zhongshan Gastric Cancer; ZSGCI, Zhongshan Gastric Cancer Immunotherapy.

### GULP1 abrogated the efficacy of anti-PD-1 therapy and inhibited CD8^+^ T-cell function in GC mouse models

First, we investigated the oncogenic role of GULP1 in human GC cell lines and immunodeficient mice. GULP1 expression was significantly higher in GC cell lines compared with that in the normal gastric epithelial cell line (Fig. S2A and B). To further explore its function, we overexpressed GULP1 in AGS cells and silenced GULP1 in HGC-27 cells (Fig. S2C and D). Both in vitro and in vivo experiments demonstrated that GULP1 exerted protumorigenic effects in GC cells (Fig. S2E to J). We then assessed the role of GULP1 in resistance to anti-PD-1 immunotherapy using immunocompetent mouse models. A subcutaneous GC model was established with YTN-16 cells stably overexpressing *Gulp1* or vector control YTN-16 cells (Fig. S3A and B). Anti-PD-1 monoclonal antibody or IgG2a control was administered 5 weeks postinoculation (Fig. 2A). In the vector control group, tumor growth was markedly suppressed by anti-PD-1 treatment, whereas *Gulp1*-overexpressing tumors exhibited limited therapeutic responsiveness to anti-PD-1 blockade (Fig. 2B to D). Meanwhile, mIF staining of tumor sections revealed a pronounced reduction of CD8^+^ T-cell infiltration in *Gulp1*-overexpressing tumors, regardless of anti-PD-1 therapy (Fig. 2E). Furthermore, an orthotopic GC model was established (Fig. 2F), and treatment efficacy was monitored via in vivo optical imaging (Fig. 2G). Consistent with the subcutaneous model, *Gulp1* overexpression conferred resistance to anti-PD-1 therapy in the orthotopic GC model and shortened the OS of mice either with anti-PD-1 treatment or not (Fig. 2H and I). *Gulp1* overexpression was associated with reduced survival even in the absence of anti-PD-1 treatment, suggesting a potential tumor-promoting effect independent of immunotherapy response. Flow cytometry analysis of the orthotopic tumor further demonstrated that CD8^+^ T-cell infiltration and the cytotoxic activity, as indicated by granzyme B (Gzmb) and interferon-gamma (Ifn-γ) expression, were significantly reduced in *Gulp1*-overexpressing tumors despite anti-PD-1 treatment (Fig. 2J and K). To further validate the role of *Gulp1* in anti-PD-1 resistance, a subcutaneous GC model was established using YTN-16 cells with stable *Gulp1* knockout or the corresponding control (Fig. S3C and D). Knockout of *Gulp1* not only inhibited tumor growth but also enhanced the sensitivity of tumors to anti-PD-1 therapy (Fig. S3E to G). mIF and flow cytometry analyses revealed that *Gulp1* depletion markedly increased both the infiltration and effector function of CD8^+^ T cells within the TME (Fig. S3H to J). To determine whether this anti-tumor effect was dependent on Cd8^+^ T cells, an anti-CD8 monoclonal antibody or IgG2a was administered to GC-bearing mice. Blockade of Cd8^+^ T cells abrogated the tumor-suppressive effect mediated by *Gulp1* knockout in YTN-16 (Fig. S3K to M). Collectively, these findings support that GULP1 drives resistance to anti-PD-1 therapy by restricting the effector activity of intratumoral CD8^+^ T cells.

**Fig. 2.**
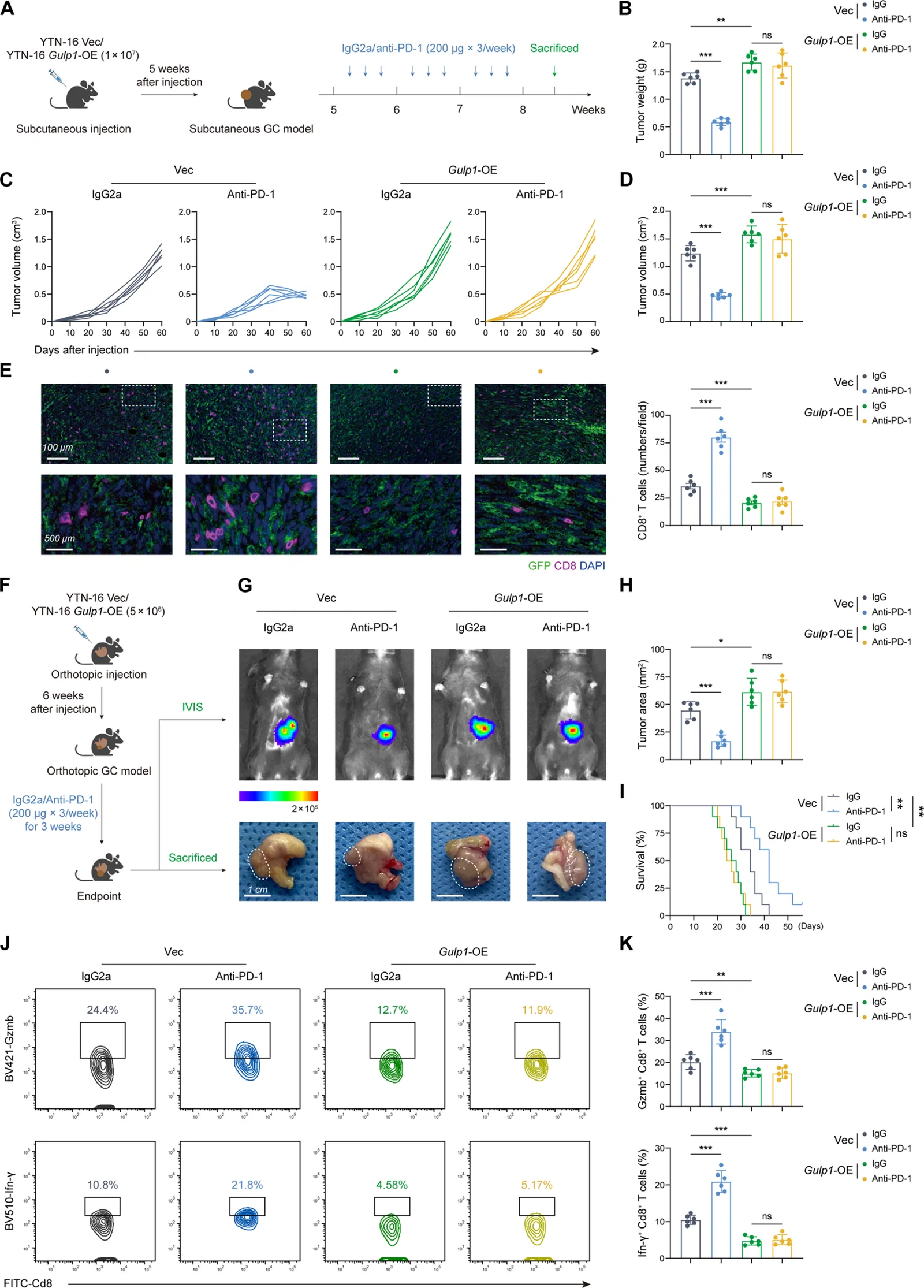
GULP1 diminishes the efficacy of anti-PD-1 treatment in a CD8^+^ T-cell-dependent manner. (A) Schematic representation of the schedule for anti-PD-1 treatment in the subcutaneous tumor mouse model. (B) Weight of subcutaneous tumor in the indicated groups (*n* = 6). (C and D) Growth curve of subcutaneous tumor and volume of subcutaneous tumor in the indicated groups at endpoint (*n* = 6). (E) mIF staining of Cd8^+^ T cells in mice tumors (boxed regions in the upper panels are shown at higher magnification in the corresponding lower panels) and quantification of the number of Cd8^+^ T cells per field. (F) Schematic representation of the schedule for anti-PD-1 treatment in the orthotopic tumor mouse model. (G) Representative images of IVIS and the orthotopic stomach tumors at the endpoint (*n* = 6). White dashed lines delineate the orthotopic gastric tumor regions. (H and I) Volume of orthotopic tumor and Kaplan–Meier survival curves for mice in the indicated groups at the endpoint (*n* = 10). (J and K) Flow cytometric analysis and quantification of Gzmb^+^ and Ifn-γ^+^ Cd8^+^ cells in the indicated groups (*n* = 6). *P < 0.05; **P < 0.01; ***P < 0.001; ns, not significant. Abbreviations: GULP1, GULP PTB domain-containing engulfment adaptor 1; Gzmb, granzyme B; Ifn-γ, interferon-γ; mIF, multiplex immunofluorescence; PD-1, programmed cell death protein 1.

### GULP1 reshaped an inhibitory TME by reprogramming CD36^+^ TAMs

To further elucidate the impact of *Gulp1* on the immune landscape of GC, we performed CyTOF to profile immune cell infiltration and functional states in the subcutaneous GC model. Clustering analysis of 42 immune markers (details shown in Table S4) identified 29 distinct cell clusters, primarily consisting of neutrophils, macrophages, Cd8^+^ T cells, Cd4^+^ T cells, B cells, natural killer cells, and dendritic cells (Fig. 3A and B and Fig. S4). Notably, *Gulp1* overexpression reduced Cd8^+^ T-cell infiltration while promoting TAM accumulation compared with control tumors (Fig. 3C). Characterization of the TAM compartment demonstrated a marked expansion of Cd36^+^ Pd-l1^+^ macrophages in tumors with enforced *Gulp1* expression (Fig. 3D and Fig. S5A and B). Functionally, *Gulp1* overexpression impaired Cd8^+^ T-cell activity: the proportion of activated (Gzmb^+^) Cd8^+^ T cells decreased, whereas exhausted Cd8^+^ T cells expressing Pd-1 were increased (Fig. 3E and Fig. S5A and C). Moreover, ssGSEA of the TCGA-STAD cohort also revealed an immunosuppressive TME in GULP1-high patients with restricted infiltration of CD8^+^ T cells and M1-type macrophages (Fig. S6). To extend these findings, patients from the scRNA-seq dataset GSE206785 were stratified into GULP1-high and GULP1-low groups. Consistent with the CyTOF results, GULP1-high patients exhibited an immunosuppressive TME with increased macrophage infiltration and reduced CD8^+^ T-cell abundance relative to GULP1-low patients (Fig. S7A). scRNA-seq analysis identified 7 macrophage subclusters, with subcluster 1 significantly enriched in GULP1-high patients (Fig. 3F and G and Fig. S7B). Functional annotation revealed that subcluster 1 expressed high levels of immunosuppressive markers, including CD163 and transforming growth factor-beta (Fig. S8A). Consistently, GC cells with enforced *Gulp1* expression attracted a greater number of macrophages in the transwell assay (Fig. S8B) and induced a shift toward an immunosuppressive phenotype, characterized by increased suppressive markers and reduced stimulatory markers (Fig. S8C). Flow cytometry indicated that *Gulp1*-overexpressing GC cells up-regulated Cd36 and Pd-l1 expression in TAMs (Fig. S8D and E) and down-regulated major histocompatibility complex class II (Mhc-ii) expression (Fig. S8F), thereby strongly suppressing T-cell proliferation and Ifn-γ production compared with controls (Fig. S8G and H). CD36 is a multifunctional scavenger receptor that facilitates the uptake of fatty acids and plays a pivotal role in lipid metabolism. Given the emerging importance of lipid metabolism within the TME, metabolic profiling analysis indicated that subcluster 1 TAMs preferentially relied on lipid metabolism, with pathways like fatty acid elongation, fatty acid degradation, and related genes significantly enriched (Fig. 3H and I). To validate the reliability of the above scRNA-seq analysis, we further analyzed an independent dataset, GSE183904, as a validation cohort. Consistent with our findings, GULP1 was predominantly expressed in malignant epithelial cells in this dataset (Fig. S9A to C). Moreover, tumors from patients with high GULP1 expression exhibited increased macrophage infiltration within the TME (Fig. S9D and E). Among macrophage subpopulations, the subcluster 0 characterized by fatty acid metabolic activity showed the most prominent enrichment in GULP1-high tumors (Fig. S9F to I), further supporting the association between tumor-intrinsic GULP1 expression and macrophage lipid metabolic reprogramming. Based on these characteristics, we speculated that CD36^+^ TAMs may represent a subcluster of lipid-associated macrophages (LAMs). Consistently, scRNA-seq analysis showed that CD36 was coexpressed with established LAM markers, including fatty acid-binding protein 5 (FABP5) and triggering receptor expressed on myeloid cells 2 (TREM2) (Fig. S10), further supporting the lipid-associated phenotype of CD36^+^ TAMs. mIF staining corroborated the above findings, revealing elevated infiltration of Cd36^+^ TAMs and reduced Cd8^+^ T-cell density in *Gulp1*-overexpressing tumors (Fig. S11A and B). Correlation analysis further demonstrated an inverse relationship between Cd36^+^ TAMs and Cd8^+^ T-cell abundance (Fig. S11C). We next examined whether this inverse association with Cd8^+^ T cells was specific to Cd36^+^ TAMs or represented a general feature of macrophages. In *Gulp1*-overexpressing tumors, Cd36^−^ macrophages were reduced and showed only a weak positive correlation with Cd8^+^ T-cell abundance (Fig. S11D and E), suggesting that the negative association with Cd8^+^ T cells was not shared by all macrophage populations. To further assess macrophage heterogeneity, we found that in GSE183904, only macrophage subcluster 0, which was characterized by high CD36 expression, exhibited a significant negative correlation with CD8^+^ T-cell abundance (Fig. S11F), whereas other macrophage subclusters did not show this relationship (Fig. S11G to L). Together, these results indicate that CD8^+^ T-cell exclusion is predominantly associated with the CD36-high TAM population rather than macrophages as a whole, supporting a specific role for CD36^+^ TAMs in establishing an immunosuppressive TME. Supporting this, TAMs co-cultured with *Gulp1*-overexpressing tumor cells exhibited an elevated neutral lipid content (detected by BODIPY 493/503 staining and BODIPY-C16 flow cytometry), higher intracellular triglyceride levels, and up-regulation of fatty acid-metabolism-related genes (Fig. 3J to L and Fig. S12A). Seahorse assays revealed that *Gulp1*-overexpressing GC cells markedly increased oxygen consumption in TAMs. This metabolic enhancement was dependent on fatty acid oxidation (FAO), as it was completely abolished by etomoxir, a carnitine palmitoyltransferase 1 (Cpt1) inhibitor (Fig. 3M and Fig. S12B). Consistently, etomoxir pretreatment also reversed the immunosuppressive phenotype (elevated Cd36 and Pd-l1 expression) of TAMs induced by *Gulp1*-overexpressing GC cells (Fig. 3N and O) and promoted T-cell cytotoxic functions (Fig. 3P and Q), indicating that FAO contributes to Gulp1-associated metabolic alterations in TAMs. Together, these findings suggest that Gulp1 promotes a shift in TAM metabolism toward FAO, which is associated with enhanced immunosuppressive functions.

**Fig. 3.**
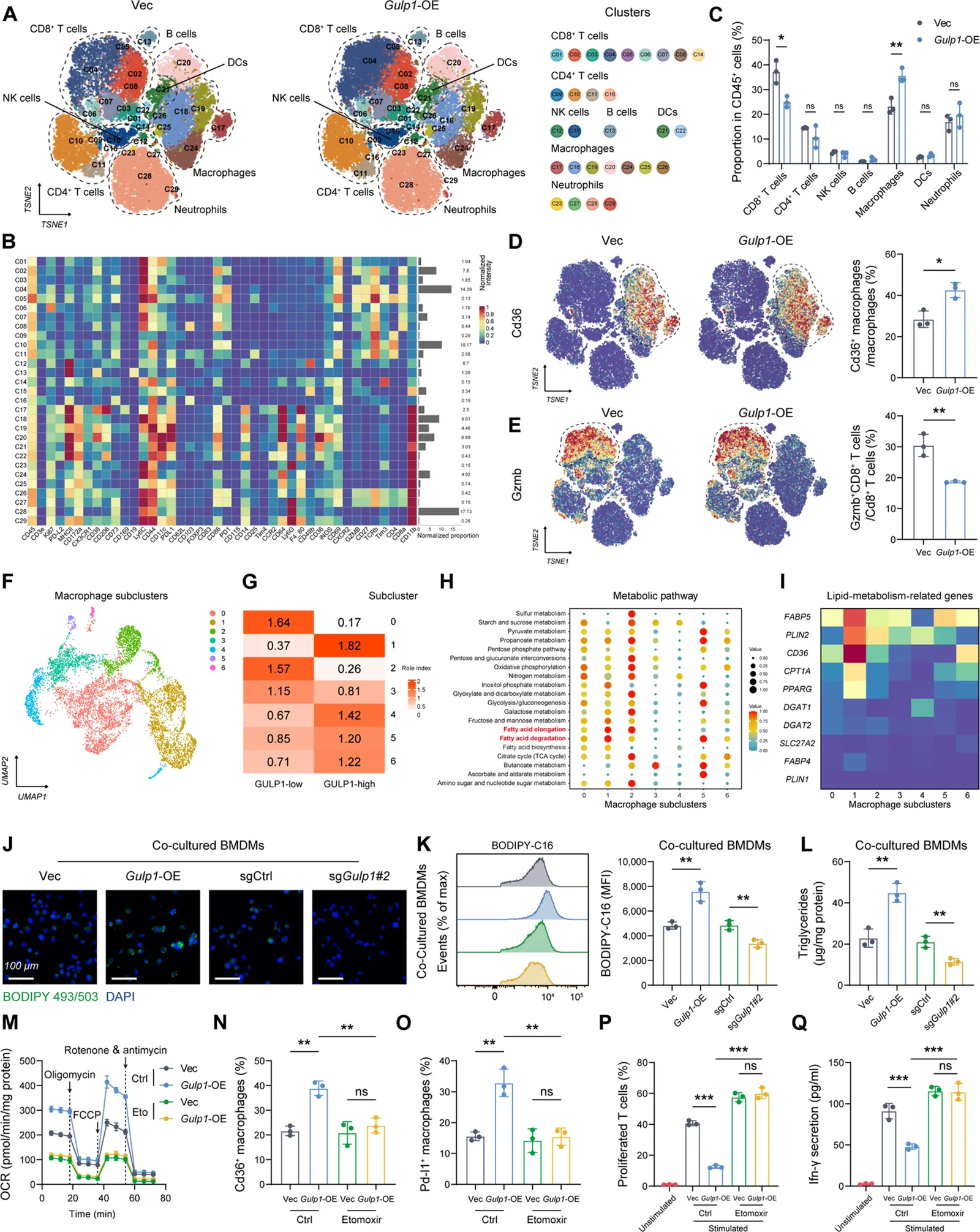
A population of lipid-loaded macrophages expressing CD36 is expanded in GULP1 overexpressed tumor where it acquires an immunosuppressive phenotype. (A) t-SNE plot depicting the subcluster of Cd45^+^ cells from subcutaneous GC tumors in the vector and *Gulp1*-OE groups (*n* = 3). (B) Heatmap of 42 markers expressed in each cell cluster. (C) Frequency of immune cells among the Cd45^+^ cells in each group. (D and E) t-SNE plots of all Cd45^+^ cells colored according to the expression levels of Cd36 and Gzmb. (F) UMAP visualization of macrophages. (G) Proportions of macrophage subclusters in GULP1-high and GULP1-low patients. (H) Metabolic pathway activity of TAM subclusters determined by scMetabolism. (I) Heatmap of lipid-metabolism-related genes in macrophage subclusters. (J) Representative images of BODIPY 493/503 staining of BMDMs co-cultured with the indicated YTN-16 cells. (K) Flow cytometry analysis of lipid uptake capacity of BMDMs co-cultured with the indicated YTN-16 cells by BODIPY FL C16. (L) Cellular triglycerides (TGs) measured in BMDMs co-cultured with the indicated YTN-16 cells. (M) OCR of BMDMs co-cultured with the indicated YTN-16 cells and BMDMs co-cultured with the indicated YTN-16 cells and treated with FAO inhibitor etomoxir (200 mmol/l). (N and O) Flow cytometry analysis of CD36 and PD-L1 expression of BMDMs in different groups. (P) CFSE assay detecting the inhibition of T-cell proliferation by BMDMs with different treatment as indicated. (Q) Ifn-γ^+^ secretion by Cd8^+^ T cells in the assay in (P), measured by ELISA. *P < 0.05; **P < 0.01; ***P < 0.001; ns, not significant. Abbreviations: BMDMs, bone-marrow-derived macrophages; BODIPY, boron–dipyrromethene difluoride; cDC, conventional dendritic cell; CFSE, carboxyfluorescein succinimidyl ester; ELISA, enzyme-linked immunosorbent assay; FAO, fatty acid oxidation; GC, gastric cancer; GULP1, GULP PTB domain-containing engulfment adaptor 1; Gzmb, granzyme B; Ifn-γ, interferon-γ; OCR, oxygen consumption rate; PD-L1, programmed death-ligand 1; t-SNE, t-distributed stochastic neighbor embedding; TAM, tumor-associated macrophage; UMAP, uniform manifold approximation and projection.

### Depletion of CD36 in macrophages impaired GULP1-induced immunosuppression in TAMs and sensitized GC to anti-PD-1 immunotherapy

To determine whether Gulp1 drives anti-PD-1 resistance by reprogramming TAMs through Cd36, we generated macrophage-specific *Cd36*-knockout mice (*Cd36*^fl/fl^*Lyz2*^Cre^) by crossing *Cd36*^fl/fl^ mice with *Lyz2*^Cre^ transgenics. TAMs isolated from *Cd36*^fl/fl^*Lyz2*^Cre^ and wild-type (WT) mice were co-cultured with GC cells overexpressing Gulp1 or the corresponding vector. In contrast to WT macrophages, TAMs from *Cd36*^fl/fl^*Lyz2*^Cre^ mice exhibited no increase in lipid metabolic activity following co-culture with *Gulp1*-overexpressing GC cells. The expression of fatty acid-metabolism-related genes, as well as lipid accumulation (assessed by BODIPY 493/503 staining and BODIPY-C16 flow cytometry), intracellular triglyceride content, and OCR, remained comparable to that in controls (Fig. 4A to E). Moreover, the Gulp1-induced immunosuppressive phenotype was abrogated in macrophage-specific *Cd36*-knockout mice, as both immunosuppressive and activation markers in TAMs were indistinguishable from those in control GC co-cultures (Fig. 4F and G). Consistently, *Gulp1*-overexpressing GC cells lost their ability to inhibit T-cell proliferation and Ifn-γ production in the absence of macrophage Cd36 (Fig. 4H and I). Functionally, macrophage-specific deletion of *Cd36* attenuated the protumor effects mediated by *Gulp1* overexpression, evidenced by markedly reduced tumor growth in *Cd36*^fl/fl^*Lyz2*^Cre^ mice compared with that in WT controls (Fig. 4J and K). Importantly, the loss of *Cd36* in macrophages rendered *Gulp1*-overexpressing tumors more responsive to anti-PD-1 therapy (Fig. 4J and K). Flow cytometry revealed that combined *Cd36* deletion and PD-1 blockade enhanced infiltration of Gzmb^+^ and Ifn-γ^+^ Cd8^+^ T cells (Fig. 4L and M). mIF staining further confirmed that *Cd36*^fl/fl^*Lyz2*^Cre^ mice treated with anti-PD-1 exhibited an immune-activated phenotype, characterized by elevated Mhc-ii expression on macrophages and heightened cytotoxicity of Cd8^+^ T cells, as indicated by increased Gzmb expression within *Gulp1*-overexpressing tumors (Fig. S13). Collectively, these findings demonstrate that *Cd36*^+^ TAMs are indispensable for Gulp1-mediated metabolic reprogramming of TAMs and resistance to anti-PD-1 therapy, highlighting the Gulp1–Cd36 axis as a potential therapeutic target in GC.

**Fig. 4.**
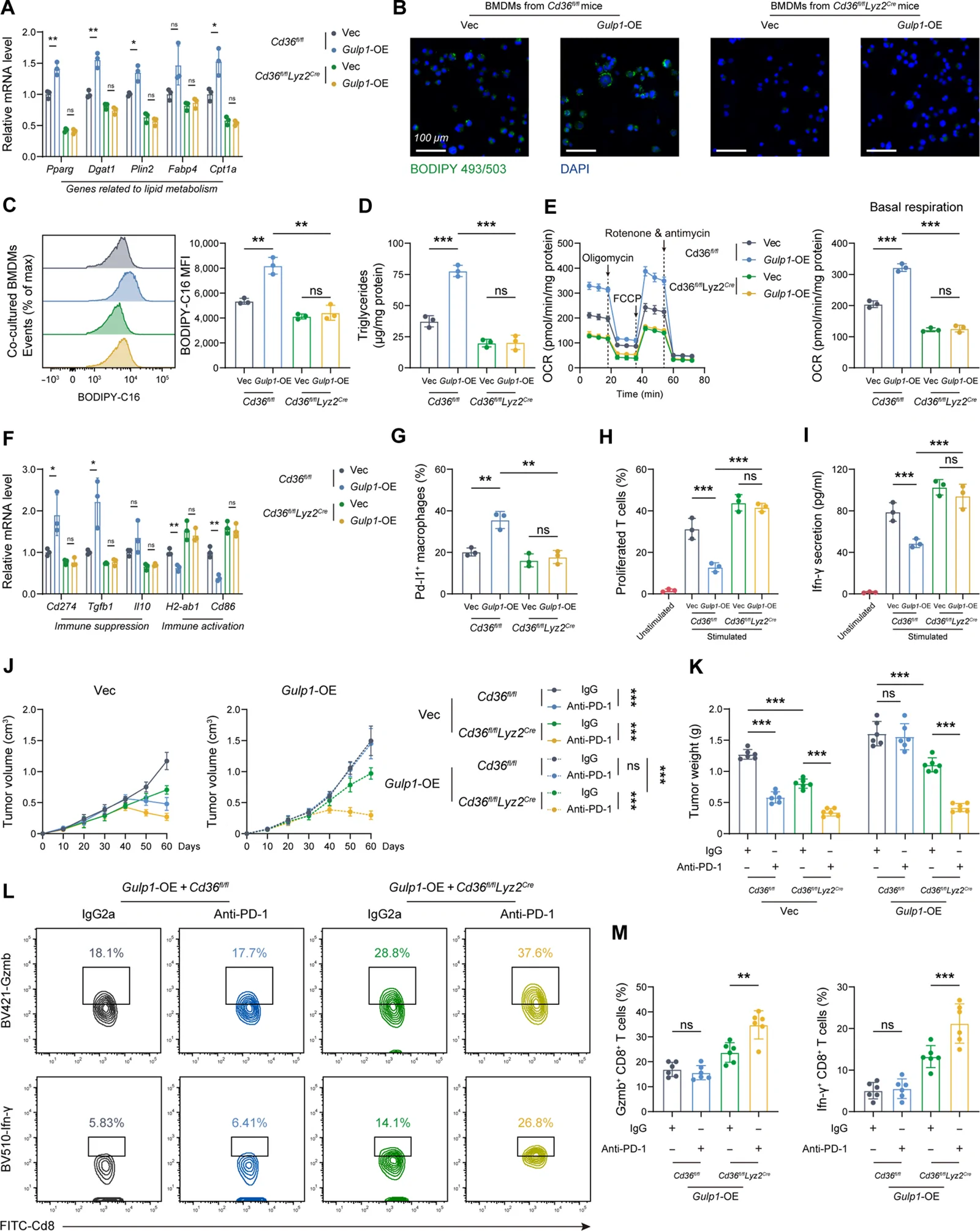
Conditional knockout of CD36^+^ macrophages abolished the anti-PD-1 resistance mediated by GULP1. (A) Lipid-metabolism-related gene expression in BMDMs extracted from *Cd36*^fl/fl^*Lyz2*^Cre^ mice and their control littermate mice co-cultured with the indicated YTN-16 cells. (B) Representative images of BODIPY 493/503 staining of *Cd36*^−/−^ and wild-type BMDMs co-cultured with the indicated YTN-16 cells. (C) Flow cytometry analysis of the lipid uptake capacity of *Cd36*^−/−^ and wild-type BMDMs co-cultured with the indicated YTN-16 cells by BODIPY FL C16. (D) TG measured in *Cd36*^−/−^ and wild-type BMDMs co-cultured with the indicated YTN-16 cells. (E) OCR of CD36^−/−^ and wild-type BMDMs co-cultured with the indicated YTN-16 cells. (F) Immune suppression- and activation-related gene expression in *Cd36*^−/−^ and wild-type BMDMs co-cultured with the indicated YTN-16 cells. (G) Flow cytometry analysis of the PD-L1 expression of *Cd36*^−/−^ and wild-type BMDMs co-cultured with the indicated YTN-16 cells. (H) CFSE assay detecting the inhibition of T-cell proliferation by BMDMs with different treatments as indicated. (I) Ifn-γ^+^ secretion by Cd8^+^ T cells in the assay in (H), measured by ELISA. (J and K) Growth curve and weight of subcutaneous tumor in the indicated groups (*n* = 6). (L and M) Flow cytometric analysis and quantification of Gzmb^+^ and Ifn-γ^+^ Cd8^+^ cells in the indicated groups (*n* = 6). *P < 0.05; **P < 0.01; ***P < 0.001; ns, not significant. Abbreviations: BMDMs, bone-marrow-derived macrophages; Cpt1a, carnitine palmitoyltransferase 1A; CFSE, carboxyfluorescein succinimidyl ester; Dgat1, diacylglycerol *O*-acyltransferase 1; ELISA, enzyme-linked immunosorbent assay; Fabp4, fatty acid-binding protein 4; GULP1, GULP PTB domain-containing engulfment adaptor 1; Gzmb, granzyme B; Ifn-γ, interferon-γ; OCR, oxygen consumption rate; PD-1, programmed cell death protein 1; PD-L1, programmed death-ligand 1; Plin2, perilipin 2; Pparg, peroxisome proliferator-activated receptor gamma; TG, triglyceride.

### GULP1 orchestrated lipid enrichment TME to reprogram CD36^+^ immunosuppression TAMs

To delineate the cross talk between GC cells and TAMs, CellChat and metabolic signaling were analyzed. Cell–cell communication analysis using CellChat revealed differences in interaction patterns between GULP1-positive and GULP1-negative tumor cells (Fig. S14A). However, these changes were broadly distributed across multiple signaling pathways and did not identify a dominant cytokine or ligand–receptor axis (Fig. S14B) that could be prioritized for mechanistic investigation. This prompted us to explore alternative regulatory mechanisms.

We next examined whether lipid metabolic pathways influence the cross talk between tumor cells and TAMs. To characterize the lipid metabolic interplay between cancer cells and macrophage subclusters, we profiled the activity of key lipid-processing enzymes and transporters using scRNA-seq data from the GSE183904 dataset. This analysis revealed that macrophages, specifically subcluster 1, exhibited markedly stronger lipid metabolic communication with GULP1^+^ tumor cells than with their GULP1^−^ counterparts (Fig. 5A), indicating that lipid metabolism constitutes a major axis of interaction in the GULP1-high context. KEGG pathway enrichment analysis revealed that GULP1-high GC cells were markedly enriched in lipid metabolic processes, including unsaturated fatty acid biosynthesis (Fig. 5B). Consistently, GULP1^+^ tumor cells displayed markedly elevated expression of fatty acid synthase (*FASN*), elongation enzymes (*ELOVL1* and *ELOVL2*), and desaturases (*FADS1* and *FADS2*) compared with other cell populations (Fig. 5C and D), suggesting that tumor cells act as the major lipid supplier within the TME. BODIPY staining confirmed that *Gulp1* overexpression enhanced intracellular fatty acid accumulation in GC cells (Fig. 5E). Moreover, GC–MS of culture media demonstrated that *Gulp1*-overexpressing GC cells contained more extracellular fatty acid in culture media (Fig. 5F). In a BODIPY-labeled co-culture system, knockdown of *Fasn* in GC cells abrogated the Gulp1-mediated increases in fatty acid synthesis and lipid transfer to macrophages (Fig. 5G and H). Correspondingly, *Fasn* silencing in GC cells reversed the heightened lipid metabolic activity observed in *Gulp1*-overexpressing co-cultured TAMs, as reflected by diminished lipid accumulation (BODIPY staining and BODIPY-C16 flow cytometry), reduced expression of lipid-metabolism-associated genes, and decreased FAO rates (Fig. 5I to L). In addition, the proportion of Cd36^+^ TAMs markedly declined when *Fasn* was silenced in *Gulp1*-overexpressing GC cells (Fig. S15A). Functionally, *Fasn* knockdown reversed the Gulp1-induced up-regulation of immune inhibitory markers in TAMs, while immune activation markers were restored (Fig. 5M and Fig. S15B and C). Consequently, T-cell proliferation and Ifn-γ secretion were significantly recovered (Fig. S15D and E). Collectively, these findings indicate that Gulp1 promotes fatty acid synthesis in GC cells, thereby supplying lipids to TAMs via CD36-dependent uptake, which fuels their metabolic reprogramming and immunosuppressive function. In subcutaneous GC models, *Fasn* depletion in *Gulp1*-overexpressing GC cells suppressed tumor growth (Fig. S16A and B), accompanied by reduced CD36^+^ TAM infiltration (Fig. 5N and Fig. S16C), and enhanced the cytotoxic activity (Gzmb^+^) of CD8^+^ T cells (Fig. 5N). Targeting the Gulp1-mediated de novo fatty acid synthesis might thus represent a promising therapeutic strategy to counteract metabolic immunosuppression and restore anti-tumor immunity in GC.

**Fig. 5.**
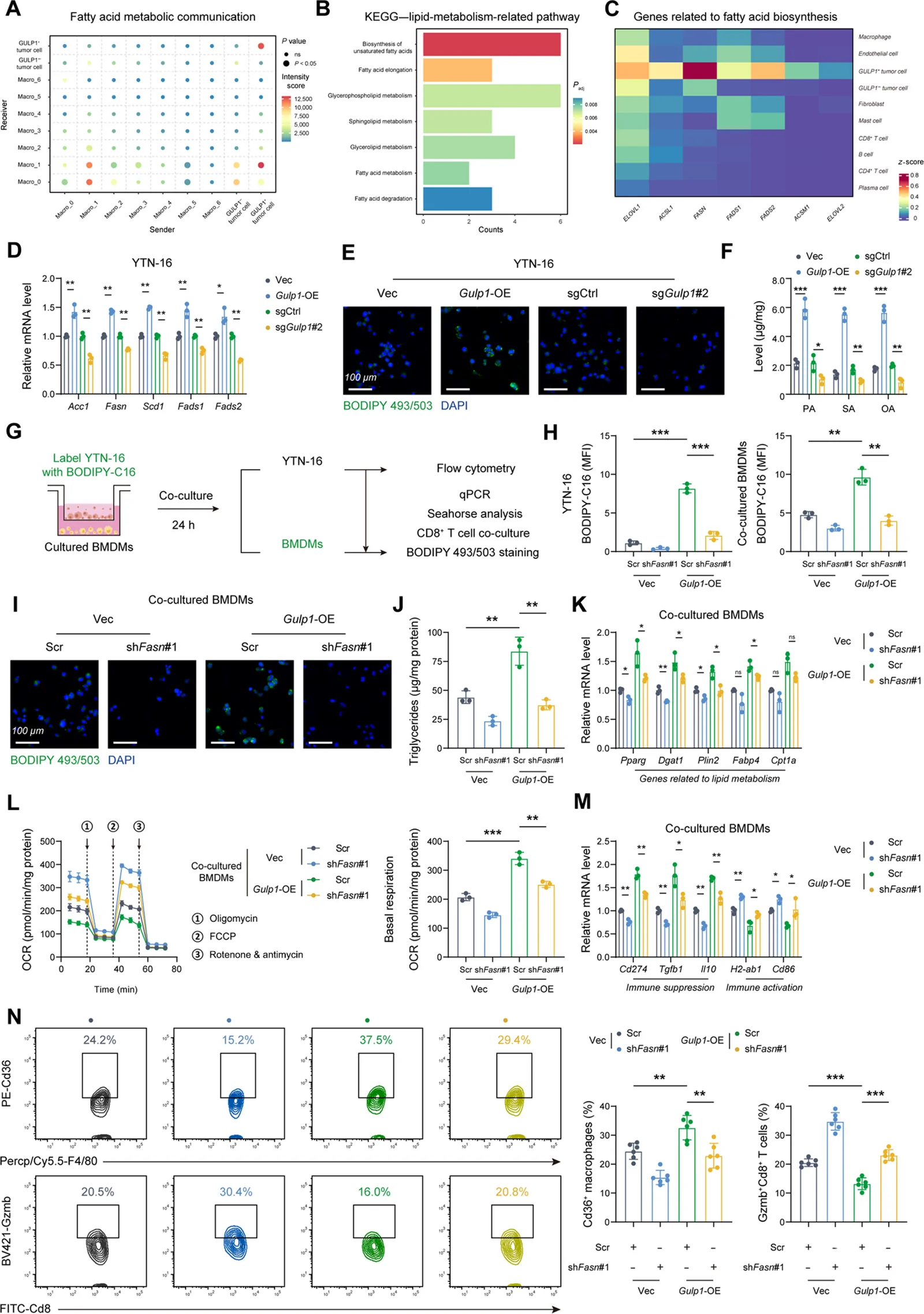
GULP1 promoted tumor fatty acid production driving CD36^+^ lipid-loaded TAMs metabolically reprogramming to exert immunosuppressive function. (A) Lipid metabolic communications between macrophage subclusters and tumor cells in the human GC TME. (B) KEGG analysis of enriched signaling pathway enriched in GULP1-high-expression GC cells. (C) Heatmap of genes related to fatty acid biosynthesis in different cell types of GC TME. (D) Fatty acid-biosynthesis-related gene expression in the indicated YTN-16 groups. (E) Representative images of BODIPY 493/503 staining of the indicated YTN-16 cells. (F) Metabolomic profiling of long-chain UFAs in the indicated YTN-16 cells. (G and H) Schematic of the indicated YTN-16 cells co-cultured with BMDMs, evaluating BODIPY-C16 levels. (I) Representative images of BODIPY 493/503 staining of BMDMs co-cultured with the indicated YTN-16 cells. (J) TG measured in BMDMs co-cultured with the indicated YTN-16 cells. (K) Lipid-metabolism-related gene expression of BMDMs co-cultured with the indicated YTN-16 cells. (L) OCR of BMDMs co-cultured with the indicated YTN-16 cells. (M) Immune suppression- and activation-related gene expression of BMDMs co-cultured with the indicated YTN-16 cells. (N) Flow cytometric analysis and quantification of Cd36^+^F4/80^+^ TAMs and Gzmb^+^Cd8^+^ cells in the indicated groups (*n* = 6). *P < 0.05; **P < 0.01; ***P < 0.001; ns, not significant. Abbreviations: Acc1, acetyl-CoA carboxylase 1; BMDMs, bone-marrow-derived macrophages; Fasn, fatty acid synthase; Fads1, fatty acid desaturase 1; Fads2, fatty acid desaturase 2; GC, gastric cancer; GULP1, GULP PTB domain-containing engulfment adaptor 1; KEGG, Kyoto Encyclopedia of Genes and Genomes; OA, oleic acid; OCR, oxygen consumption rate; PA, palmitic acid; SA, stearic acid; Scd1, stearoyl-CoA desaturase 1; TAMs, tumor-associated macrophages; TG, triglyceride; TME, tumor microenvironment; UFAs, unsaturated fatty acids.

### GULP1 promoted SREBP1-activated lipogenesis by amplifying INSIG2 palmitoylation

To elucidate how GULP1 enhances lipogenesis in GC cells, we conducted LC–MS/MS to identify GULP1-associated proteins potentially involved in lipid metabolic regulation. Among 145 candidates, insulin-induced gene 2 (INSIG2) emerged as a notable interactor due to its critical role in controlling lipid biosynthesis (Fig. 6A). Co-IP assays confirmed the interaction between GULP1 and INSIG2 (Fig. 6B). Functionally, GULP1 disrupted the INSIG2–SREBP cleavage-activating protein (SCAP) complex, facilitating SCAP translocation from the endoplasmic reticulum (ER) to the Golgi apparatus and subsequent nuclear accumulation of sterol regulatory element-binding protein 1 (SREBP1) (Fig. 6C and D and Fig. S17A to C). This translocation resulted in the transcriptional activation of SREBP1 downstream lipogenic genes, including acetyl-CoA carboxylase 1 (*ACC1*), fatty acid synthase (*FASN*), and stearoyl-CoA desaturase 1 (*SCD1*) (Fig. 6E). INSIG2 is an ER-resident protein that anchors the SREBP–SCAP complex in the ER, thereby restraining SREBP activation and lipid/cholesterol biosynthesis [28]. Previous studies have shown that phosphorylation of INSIGs at Ser207 (INSIG1) or Ser151 (INSIG2) disrupts sterol binding and the INSIG–SCAP interaction, allowing SREBP activation [29,30]. However, our findings revealed that GULP1 modulation did not alter the phosphorylation status of INSIG2 (Fig. S18). We therefore explored alternative posttranslational modifications that might mediate this effect. Neither acetylation nor lactylation of INSIG2 was influenced by GULP1 expression, whereas its palmitoylation level was markedly affected (Fig. 6F and G and Fig. S18). Sequence analysis identified 7 evolutionarily conserved cysteine residues within INSIG2. To determine the palmitoylation site, we generated a series of INSIG2 cysteine-to-serine mutants (Fig. S19). Complete substitution of all cysteines (INSIG2 C-all-S) abolished INSIG2 palmitoylation (Fig. 6H). Stepwise reversion of individual serine residues back to cysteine pinpointed Cys14 as the principal palmitoylation site (Fig. 6H). To directly assess the role of INSIG2 palmitoylation in GULP1-driven metabolic remodeling, we performed rescue experiments by reconstituting *INSIG2*-silenced cells with either WT *INSIG2* or a palmitoylation-deficient C14S mutant. While *INSIG2*-WT robustly restored SREBP1 nuclear localization and lipogenic gene expression, the C14S mutant exhibited only partial rescue, indicating that palmitoylation at Cys14 is required for the full activation of the SREBP1 pathway (Fig. S20A and B). Consistent with this, *Insig2*-WT more effectively reinstated lipid metabolic activity (Fig. S20C and D) and the immunosuppressive phenotype of TAMs, whereas the C14S mutant showed attenuated effects (Fig. S20E and F). Functionally, *Insig2*-WT reconstitution resulted in stronger suppression of Cd8^+^ T-cell proliferation and Ifn-γ production compared with the mutant (Fig. S20G and H). These results support a model in which INSIG2 palmitoylation acts as a critical, although not exclusive, regulatory determinant of the GULP1–INSIG2–SREBP1 axis. Importantly, mutation of Cys14 nullified GULP1’s ability to regulate INSIG2 palmitoylation (Fig. 6I and J) and to modulate the interaction between INSIG2 and SCAP (Fig. 6K and L). Likewise, results indicated that this mutation abolished GULP1-mediated SREBP1 nuclear translocation and the subsequent up-regulation of lipogenic genes (Fig. 6M and N). Collectively, these findings demonstrate that GULP1 promotes the dissociation of the INSIG2–SCAP complex by enhancing INSIG2 palmitoylation at Cys14, thereby activating SREBP1 and driving lipid biosynthesis in GC cells.

**Fig. 6.**
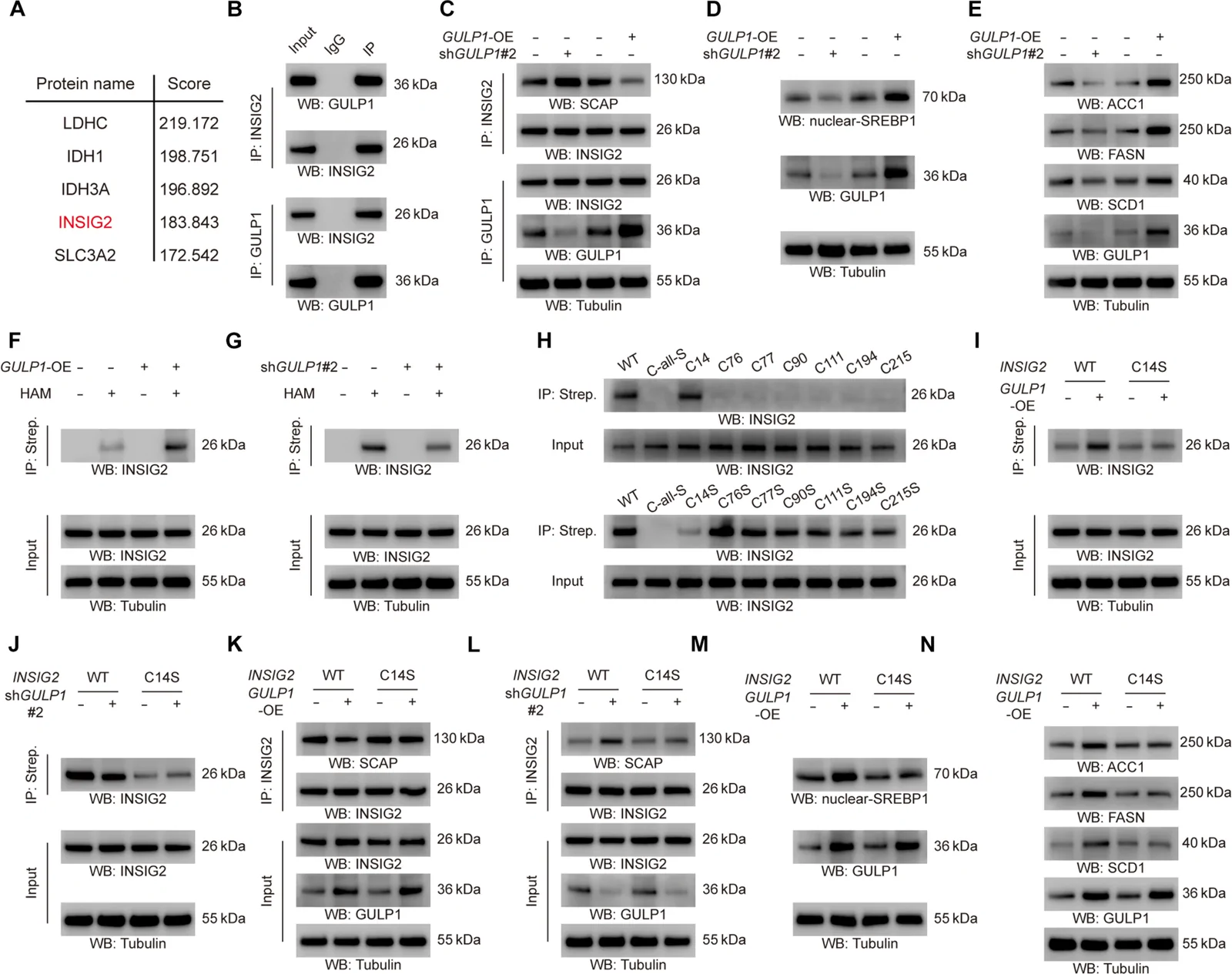
GULP1 enhances INSIG2 palmitoylation to promote SREBP1 activation for lipogenesis. (A) Five potential proteins interact with GULP1 based on protein identification confidence score. (B) Coprecipitation of endogenous GULP1 with INSIG2 from GC cells. (C) Coprecipitation of endogenous GULP1 with INSIG2 and INSIG2 with SCAP in the indicated GC cells. (D) Western blotting analysis of cleaved nuclear SREBP1 in the indicated GC cells. (E) Western blotting analysis of lipogenesis proteins in the indicated GC cells. (F and G) Western blotting analysis of INSIG2 palmitoylation by ABE assay in the indicated GC cells. (H) Western blotting analysis of INSIG2 palmitoylation by ABE assay in GC cells transfected with plasmids encoding WT INSIG2-Flag or its indicated mutants. (I and J) Western blotting analysis of INSIG2 palmitoylation by ABE assay in the indicated GC cells. (K and L) Coprecipitation of INSIG2 with SCAP in the indicated GC cells. (M) Western blotting analysis of nuclear SREBP1 in the indicated GC cells. (N) Western blotting analysis of lipogenesis proteins in the indicated GC cells. Abbreviations: ABE, acyl–biotin exchange; GC, gastric cancer; GULP1, GULP PTB domain-containing engulfment adaptor 1; IDH1, isocitrate dehydrogenase 1; IDH3A, isocitrate dehydrogenase 3 catalytic subunit alpha; INSIG2, insulin-induced gene 2; LDHC, lactate dehydrogenase C; SCAP, SREBP cleavage-activating protein; SLC3A2, solute carrier family 3 member 2; SREBP1, sterol regulatory element-binding protein 1.

### GULP1 facilitated INSIG2 palmitoylation via recruiting zDHHC palmitoyltransferase 8

Given that GULP1 functions as an adaptor protein and lacks intrinsic enzymatic activity to directly catalyze palmitoylation, we speculated that a specific palmitoyltransferase might mediate the GULP1-driven modification of INSIG2. To identify such an enzyme, we performed LC–MS/MS analysis using anti-INSIG2 immunoprecipitation to capture its binding partners. By comparing the interactomes of INSIG2-binding proteins and GULP1-binding proteins with DHHC-type palmitoyltransferase family proteins, we identified zinc finger DHHC-type palmitoyltransferase 8 (zDHHC8) as a shared interactor and a potential mediator of INSIG2 palmitoylation (Fig. 7A). Subsequent Co-IP assays confirmed that zDHHC8 physically associated with both GULP1 and INSIG2 and facilitated the palmitoylation of INSIG2 (Fig. 7B to E). Importantly, mutation of the Cys14 residue in *INSIG2* completely abolished zDHHC8-induced palmitoylation (Fig. 7F and G), indicating that zDHHC8 specifically modifies INSIG2 at this cysteine site. Moreover, GULP1 markedly strengthened the interaction between zDHHC8 and INSIG2, thereby amplifying zDHHC8-dependent palmitoylation of INSIG2 (Fig. 7H and I). To assess the functional contribution of zDHHC8, we performed knockdown experiments in the GULP1–INSIG2–SREBP1 axis. *zDHHC8* depletion significantly reduced SREBP1 activation and lipogenic gene expression (Fig. S21A and B), accompanied by decreased lipid accumulation and impaired FAO activity in TAMs (Fig. S21C and D). Consistently, *zdhhc8* knockdown diminished the proportion of Cd36^+^ and Pd-l1^+^ macrophages (Fig. S21E and F) and alleviated TAM-mediated suppression of Cd8^+^ T-cell function (Fig. S21G and H). These findings indicate that zDHHC8 is functionally required for the activation of the lipogenic and immunosuppressive program downstream of GULP1. Collectively, these findings demonstrate that GULP1 promotes INSIG2 palmitoylation indirectly by recruiting zDHHC8, establishing a GULP1–zDHHC8–INSIG2 signaling axis critical for lipid metabolic activation in GC cells.

**Fig. 7.**
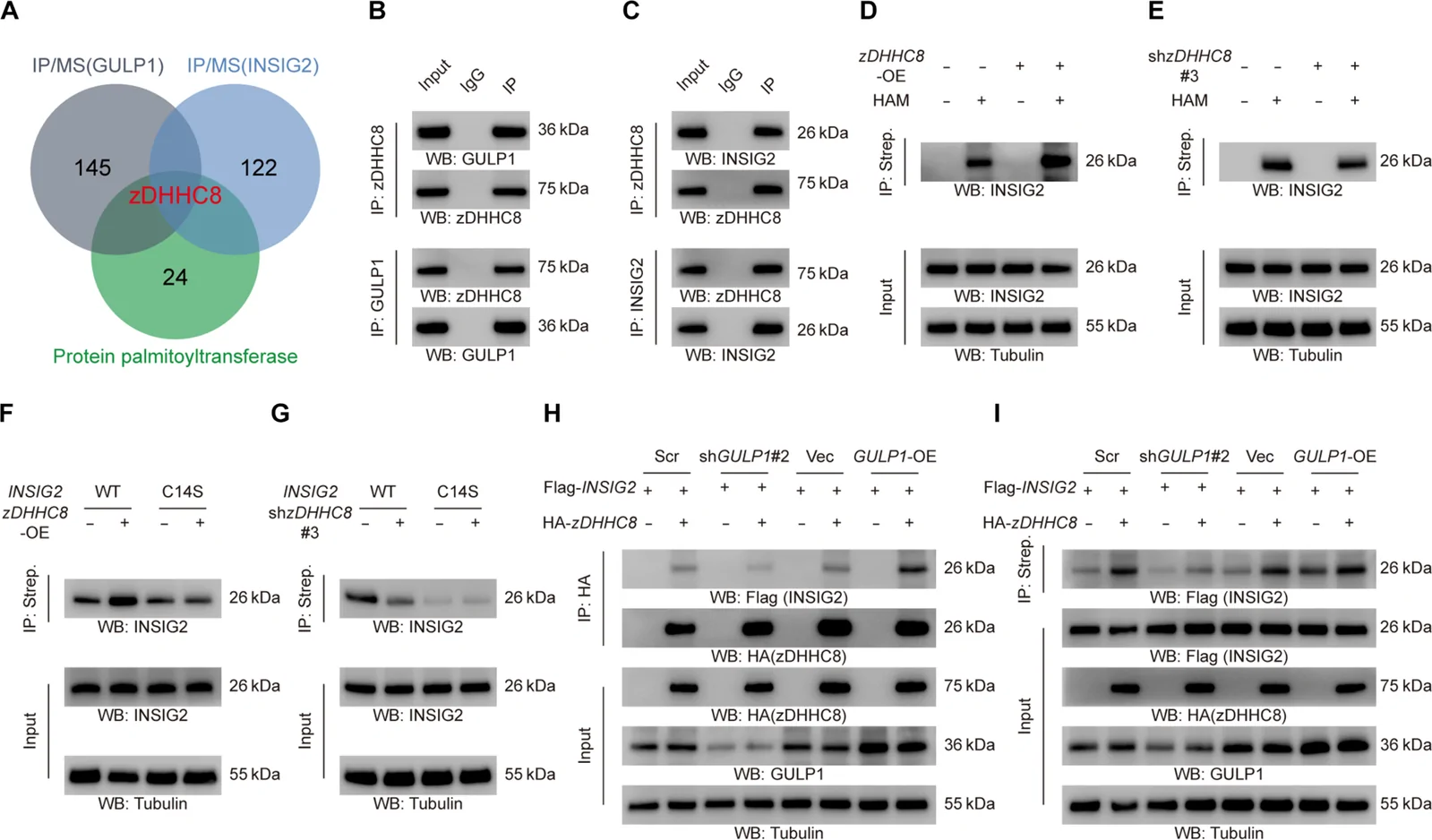
GULP1 potentiates the interaction of INSIG2 and zDHHC8 to promote INSIG2 palmitoylation. (A) Interacting proteins associated with INSIG2 and GULP1 overlapping with DHHC family proteins shown by a Venn diagram. (B) Coprecipitation of endogenous GULP1 with zDHHC8 from GC cells. (C) Coprecipitation of endogenous INSIG2 with zDHHC8 from GC cells. (D to G) Western blotting analysis of INSIG2 palmitoylation by ABE assay in the indicated GC cells. (H) Coprecipitation of zDHHC8 with INSIG2 in the indicated GC cells. (I) Western blotting analysis of INSIG2 palmitoylation by ABE assay in the indicated GC cells. Abbreviations: ABE, acyl–biotin exchange; DHHC, aspartate-histidine-histidine-cysteine; GC, gastric cancer; GULP1, GULP PTB domain-containing engulfment adaptor 1; INSIG2, insulin-induced gene 2; zDHHC8, zinc finger DHHC-type palmitoyltransferase 8.

### GA restrained GC progression by preventing GULP1 and INSIG2 interaction

Given the absence of commercially available GULP1 inhibitors, we employed a structure-based high-throughput virtual screening of 25,361 compounds to identify potential candidate small-molecule modulators of the GULP1 axis (Fig. S22A). Based on the predicted binding affinity, 40 top-ranking compounds were selected for surface plasmon resonance analysis. The results indicated that GA exhibited the highest binding affinity to the GULP1 protein, forming hydrogen bonds at *ASN-92*, *GLN-94*, *HIE-96*, *ARG-97*, *LYS-116*, *ASP-117*, and *ARG-163* (Fig. S22B). Co-IP analyses revealed that the binding of GULP1 to INSIG2 gradually decreased after the addition of increasing concentrations of GA (Fig. 8A). Administration of GA blocked the interaction between INSIG2 and zDHHC8 and the palmitoylation of INSIG2, therefore inhibiting SCAP translocation to the Golgi apparatus from the ER (Fig. 8B and C and Fig. S23A and B). Results indicated that GA promoted SREBP1 nucleus translocation and activated lipogenesis (Fig. 8D and E and Fig. S23C). BODIPY staining confirmed that GA treatment inhibited intracellular fatty acid accumulation in YTN-16 (Fig. 8F). Moreover, GC–MS of culture media demonstrated that GA treatment decreased extracellular fatty acid in the culture media of control YTN-16 (Fig. 8G). In the BODIPY-labeled co-culture system, GA treatment abrogated fatty acid synthesis in YTN-16 and its lipid transfer to TAMs (Fig. 8H). Correspondingly, GA-treated YTN-16 constrained the lipid metabolic activity observed in control YTN-16 co-cultured TAMs, as reflected by diminished lipid accumulation (BODIPY staining), decreased FAO rates, and reduced expression of immunosuppressive genes (Fig. 8I to K). Notably, the GA-driven changes were entirely lost following *Gulp1* knockout in YTN-16 cells (Fig. 8F to K), demonstrating that GA’s regulation of de novo fatty acid biosynthesis and the TME in GC is contingent on *Gulp1*. In the subcutaneous GC model, GA treatment suppressed tumor growth; however, this effect was not further enhanced in the *Gulp1*-knockout group (Fig. 8L), indicating its on-target specificity in vivo. mIF revealed that GA treatment decreased the infiltration of Cd36^+^ TAMs (Fig. 8M). Flow cytometry further proved that GA treatment restrained the infiltration of Cd36^+^ TAMs and enriched Gzmb^+^Cd8^+^ T cells (Fig. 8N). Moreover, GA treatment could efficiently inhibit de novo fatty acid synthesis and metabolically reshape TAMs even under *Gulp1* overexpression conditions in vitro (Fig. S24). In the subcutaneous GC model, GA treatment significantly reduced tumor volume and weight, decreased Cd36^+^ immunosuppressive macrophage infiltration while increasing Mhc-ii^+^ macrophages, and concomitantly enhanced the proportion of Gzmb^+^Cd8^+^ T cells, particularly in *Gulp1*-overexpressing tumors (Fig. S25). Generally, these results identified GA as a promising GULP1-targeting compound that disrupted the GULP1/zDHHC8/INSIG2/SREBP1 axis to attenuate tumor progression in GC with high sensitivity and specificity.

**Fig. 8.**
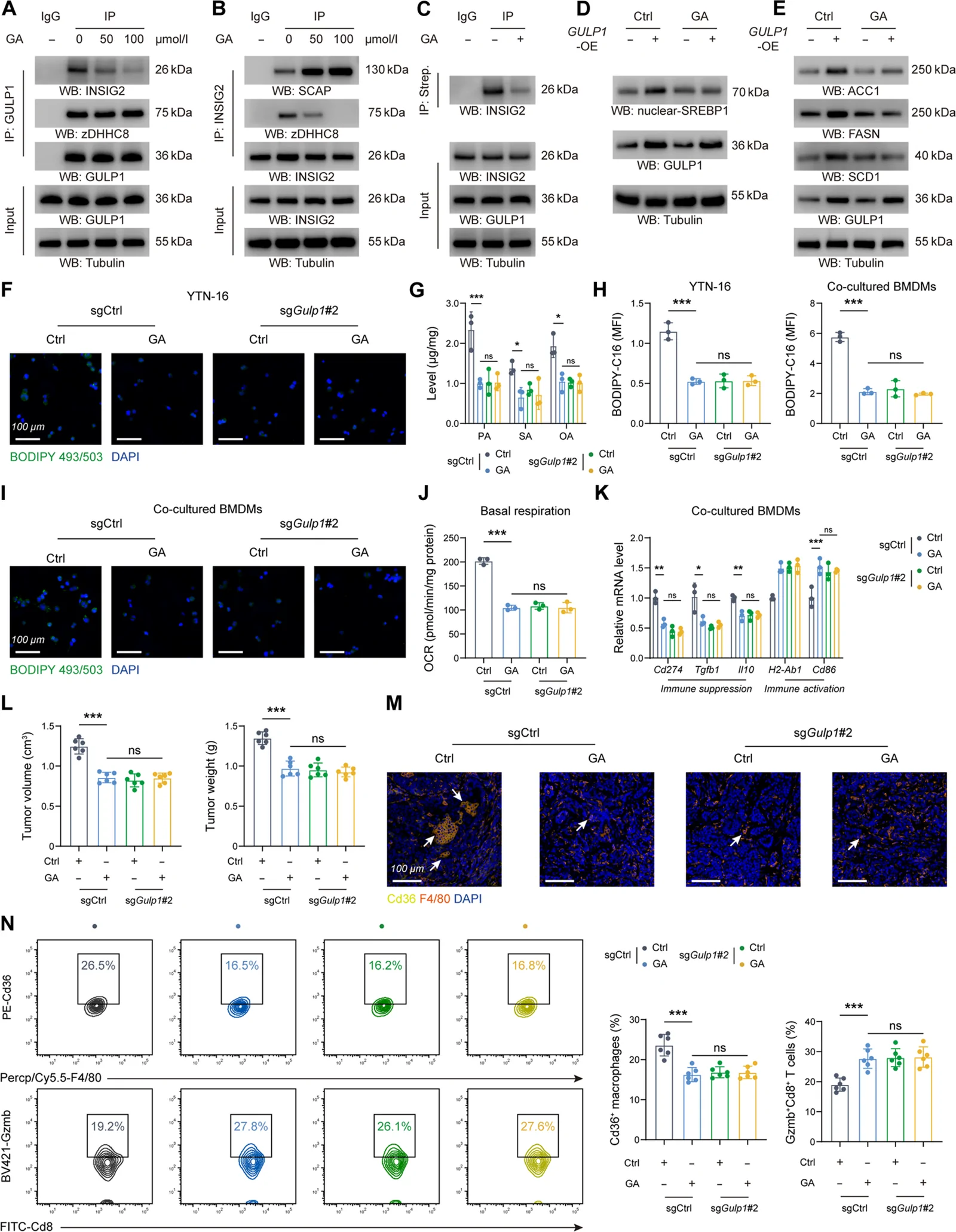
Glycyrrhizic acid, a small-molecule inhibitor of GULP1, suppresses tumor progression via boosting immune response in GC. (A) Coprecipitation of GULP1 with INSIG2 and zDHHC8 in GC cells treated with different concentrations of GA. (B) Coprecipitation of INSIG with zDHHC8 and SCAP in GC cells treated with different concentrations of GA. (C) Western blotting analysis of INSIG2 palmitoylation by ABE assay in the indicated GC cells. (D) Western blotting analysis of nuclear SREBP1 in the indicated GC cells. (E) Western blotting analysis of lipogenesis proteins in the indicated GC cells. (F) Representative images of BODIPY 493/503 staining of the indicated YTN-16 cells. (G) Metabolomic profiling of long-chain UFAs in the indicated YTN-16 cells. (H) BODIPY-C16 levels in the indicated YTN-16 cells and in BMDMs co-cultured with the respective YTN-16 cells. (I) Representative images of BODIPY 493/503 staining of BMDMs co-cultured with the indicated YTN-16 cells. (J) Basal respiration of BMDMs co-cultured with the indicated YTN-16 cells according to OCR. (K) Immune suppression- and activation-related gene expression of BMDMs co-cultured with the indicated YTN-16 cells. (L) Volume and weight of subcutaneous tumors in the indicated groups (*n* = 6). (M) mIF analysis of Cd36^+^F4/80^+^ immune cells in subcutaneous tumor tissues. Representative cells are indicated by arrows. (N) Flow cytometric analysis and quantification of Cd36^+^F4/80^+^ TAMs and Gzmb^+^Cd8^+^ cells in the indicated tumor groups (*n* = 6). *P < 0.05; **P < 0.01; ***P < 0.001; ns, not significant. Abbreviations: ABE, acyl–biotin exchange; BMDMs, bone-marrow-derived macrophages; GA, glycyrrhizic acid; GC, gastric cancer; GULP1, GULP PTB domain-containing engulfment adaptor 1; Gzmb, granzyme B; H2-Ab1, histocompatibility 2, class II antigen A, beta 1; Il10, interleukin 10; INSIG2, insulin-induced gene 2; mIF, multiplex immunofluorescence; OCR, oxygen consumption rate; SCAP, SREBP cleavage-activating protein; SREBP1, sterol regulatory element-binding protein 1; TAMs, tumor-associated macrophages; Tgfb1, transforming growth factor beta 1; UFAs, unsaturated fatty acids; zDHHC8, zinc finger DHHC-type palmitoyltransferase 8.

### Targeting GULP1 with GA augmented the efficacy of anti-PD-1 therapy in GC

The above observations prompted us to investigate whether pharmacologic inhibition of GULP1 could potentiate the therapeutic response to anti-PD-1 therapy in GC (Fig. 9A). In subcutaneous GC models, administration of GA markedly reduced tumor growth compared with vehicle control (Fig. 9B to D). Moreover, combined treatment with GA and anti-PD-1 antibody resulted in a substantially greater suppression of tumor progression than anti-PD-1 monotherapy (Fig. 9B to D). mIF analyses revealed that the combination treatment significantly diminished the infiltration of Cd36^+^ TAMs while enhancing the abundance of Gzmb^+^Cd8^+^ T cells within the TME (Fig. 9E to G). To further validate these findings, an orthotopic GC model was employed. Consistent with the subcutaneous results, GA combined with anti-PD-1 therapy markedly delayed tumor growth (Fig. 9H and I) and significantly prolonged the survival of tumor-bearing mice (Fig. 9J) without inducing detectable hepatic or renal toxicity (Fig. 9K). Notably, dual treatment elicited a robust anti-tumor immune response, characterized by a marked reduction of CD36^+^ immunosuppressive TAMs (Fig. 9L), accompanied by increased proportions of MHC-II^+^ macrophages (Fig. 9M) and enhanced infiltration of GZMB^+^ and IFN-γ^+^ CD8^+^ T cells within gastric tissues (Fig. 9N and O). Together, these results demonstrate that pharmacological inhibition of GULP1 by GA effectively enhances the efficacy of anti-PD-1 immunotherapy by remodeling the tumor immune microenvironment and restoring CD8^+^ T-cell cytotoxic function in GC. To further substantiate its therapeutic potential, we tested GA in combination with anti-PD-1 in PDOs derived from GC patients and cultured in 3D with autologous CD8^+^ T cells, and CD68^+^ macrophages. In PDOs with high GULP1 expression, GA-dependent suppression of GULP1 restored sensitivity to anti-PD-1, whereas in GULP1-low PDOs, GA markedly enhanced the anti-tumor efficacy of anti-PD-1 therapy (Fig. S26). These findings further support that targeting GULP1 represents a promising therapeutic strategy to overcome resistance and improve the efficacy of anti-PD-1 immunotherapy in GC.

**Fig. 9.**
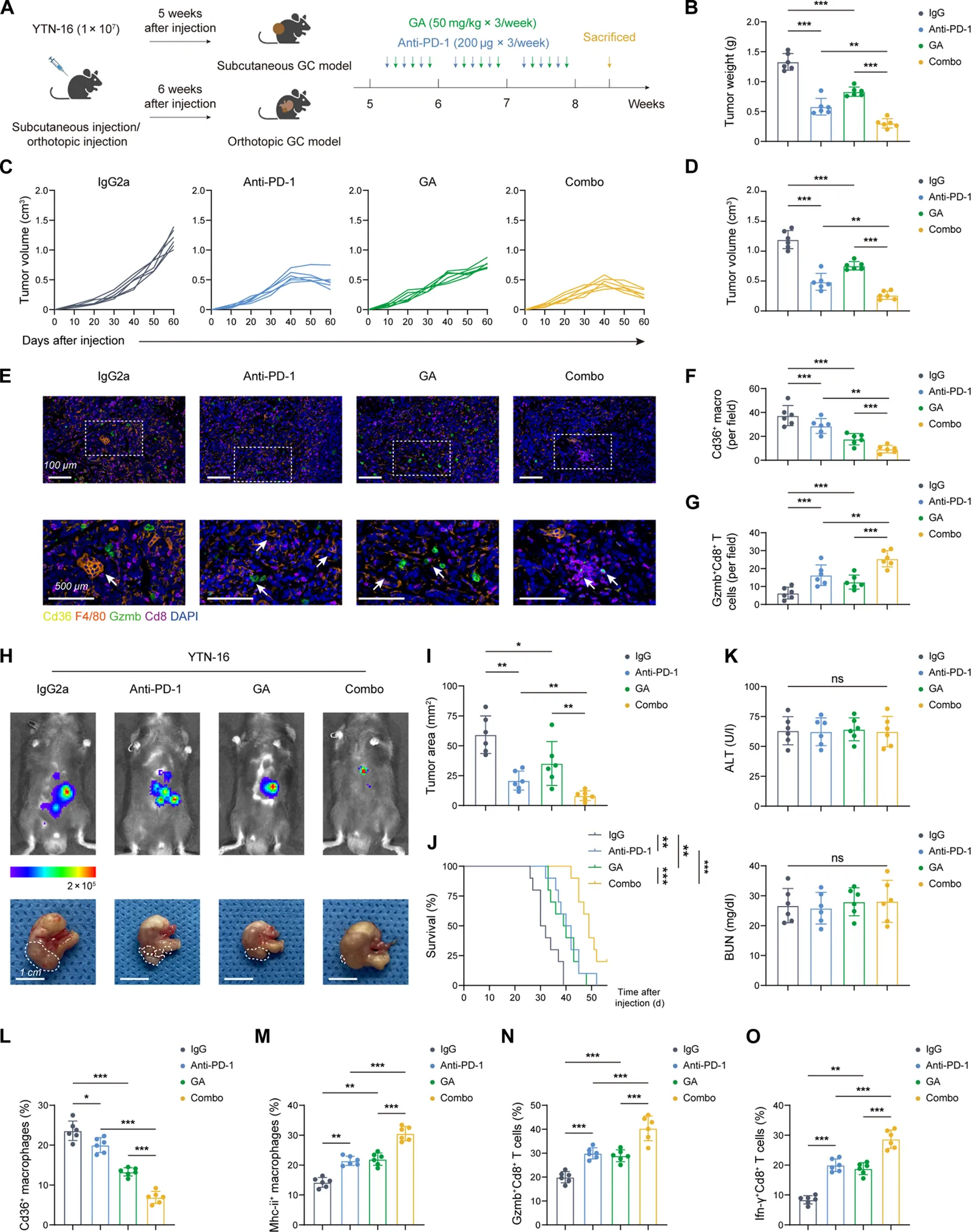
Glycyrrhizic acid enhances anti-PD-1 therapeutic efficacy by reshaping the TME in GC models. (A) Schematic representation of the schedule for anti-PD-1 and/or GA treatment in the subcutaneous and orthotopic tumor mouse model. (B) Weight of subcutaneous tumor in the indicated groups (*n* = 6). (C and D) Growth curve of subcutaneous tumor and volume of subcutaneous tumor in the indicated groups at the endpoint (*n* = 6). (E to G) mIF staining of Cd36^+^ TAMs and Gzmb^+^Cd8^+^ T cells in mice tumors, as indicated by arrows, and quantification of the numbers of Cd36^+^ TAMs and Gzmb^+^Cd8^+^ T cells per field. (H) Representative images of IVIS and the orthotopic stomach tumors at the endpoint (*n* = 6). White dashed lines delineate the orthotopic gastric tumor regions. (I and J) Volume of orthotopic tumor (*n* = 6) and Kaplan–Meier survival curves for mice in the indicated groups (*n* = 10) at the endpoint. (K) Liver and kidney functions of mice with tumors in each group at the study endpoint (*n* = 6). (L and M) Quantification of Cd36^+^ and Mhc-ii^+^ TAMs from flow cytometric analysis in the indicated groups (*n* = 6). (N and O) Quantification of Gzmb^+^ and Ifn-γ^+^ Cd8^+^ cells from flow cytometric analysis in the indicated groups (*n* = 6). *P < 0.05; **P < 0.01; ***P < 0.001; ns, not significant. Abbreviations: ALT, alanine aminotransferase; BUN, blood urea nitrogen; GA, glycyrrhizic acid; GC, gastric cancer; Gzmb, granzyme B; mIF, multiplex immunofluorescence; Mhc-ii, major histocompatibility complex class II; PD-1, programmed cell death protein 1; TAMs, tumor-associated macrophages; TME, tumor microenvironment.

## Discussion

In this study, we identified GULP1 as a tumor-intrinsic regulator associated with anti-PD-1 resistance in GC. Elevated GULP1 expression was associated with poor clinical outcomes and reduced CD8^+^ T-cell-mediated anti-tumor responses in GC patients. Functionally, tumor-intrinsic GULP1 promoted resistance to PD-1 blockade, whereas targeting GULP1 restored anti-tumor immune responses. Mechanistically, GULP1 reshaped the tumor immune microenvironment through lipid metabolic reprogramming of CD36^+^ TAMs, thereby impairing cytotoxic T-cell activity. These findings reveal a GULP1-mediated mechanism of immune evasion and suggest that the GULP1–CD36^+^ TAM axis may represent a potential therapeutic vulnerability for improving PD-1 blockade efficacy in GC.

Previous studies have suggested that GULP1 exerts context-dependent and multifaceted functions across different diseases and pathophysiology process [31,32]. However, its specific role within the GC TME has remained largely undefined. Effective PD-1 blockade requires the presence of functionally active effector T cells within the TME [33]. Our findings reveal that GULP1 expression is inversely associated with both the infiltration and activation of CD8^+^ T lymphocytes in GC tissues from mice and humans. Moreover, enforced GULP1 expression in immunocompetent mouse models markedly impaired the therapeutic efficacy of anti-PD-1 treatment by reducing CD8^+^ T-cell infiltration and activation. Notably, we observed that GULP1 overexpression was associated with reduced survival with or without anti-PD-1 treatment, suggesting that GULP1 may exert tumor-promoting effects beyond immune-mediated resistance. This observation indicates that GULP1 functions not only as a candidate of immunotherapy response but also as a regulator of tumor progression. Supporting this notion, our in vitro and in vivo data demonstrate that GULP1 enhances tumor cell growth, consistent with a tumor-intrinsic role in promoting malignancy. In parallel, GULP1 reshapes the TME by inducing lipid-dependent immunosuppressive TAM phenotypes, thereby impairing CD8^+^ T-cell function and contributing to resistance to anti-PD-1 therapy. Collectively, these findings support a model in which GULP1 drives tumor progression through both predominantly tumor-intrinsic mechanisms and modulation of the TME.

We found that GULP1 promotes the enrichment of TAMs within the GC TME. TAMs are a class of immunosuppressive cells whose accumulation has been consistently linked to poor clinical outcomes, impaired cytotoxic T-cell function, and resistance to anti-PD-1 therapy [34]. Notably, GULP1 overexpression preferentially expanded a TAM subcluster with high CD36 expression. CD36, a class II scavenger receptor responsible for long-chain fatty acid uptake, has been implicated in supporting tumor growth and metastasis through enhanced lipid metabolism [35]. Elevated CD36 levels also potentiate the suppressive function of regulatory T cells and contribute to CD8^+^ T-cell exhaustion [36]. In macrophages, CD36-dependent FAO drives polarization toward an M2-like phenotype, thereby attenuating CD8^+^ T-cell cytotoxicity. Furthermore, CD36-mediated lipid accumulation up-regulates PD-L1 expression via the peroxisome proliferator-activated receptor gamma (PPARγ) signaling pathway [37]. In our study, the increase in CD36^+^ TAMs induced by GULP1 was associated with diminished CD8^+^ T-cell activation and effector function. Conversely, macrophage-specific CD36 deletion reprogrammed TAMs toward a proinflammatory phenotype and effectively restored responsiveness to anti-PD-1 therapy, underscoring the essential role of CD36^+^ TAMs in GULP1-mediated immunotherapy resistance. Of note, a recent report identified a CD36-blocking antibody, PLT012, capable of eliciting potent anti-tumor immune responses in both immunotherapy-sensitive and immunotherapy-refractory hepatocellular carcinoma models [38]. Together with our findings, these results highlight CD36 as a promising therapeutic target for reprogramming macrophages and enhancing the efficacy of ICB in GC. While our data identify CD36 as a major mediator of GULP1-driven lipid metabolic remodeling in TAMs, fatty acid uptake and utilization can occur through multiple complementary pathways. Myeloid-specific deletion of CD36 markedly attenuated lipid accumulation, FAO-associated metabolic activity, and resistance to anti-PD-1 therapy, supporting an important role for CD36 in this process. However, given the complexity of lipid handling in myeloid cells, it is unlikely that CD36 represents the sole pathway involved. Indeed, our single-cell transcriptomic analysis revealed elevated expression of alternative lipid transport and binding molecules, such as FABP4 and FABP5, in macrophage subclusters enriched in GULP1-high tumors, suggesting that additional lipid-handling mechanisms may contribute to the observed phenotype. These findings raise the possibility that residual lipid uptake and metabolic activity may persist in CD36-deficient settings through compensatory pathways, including other fatty acid transporters and scavenger receptors. Therefore, while CD36 appears to be a key mediator of GULP1-induced metabolic reprogramming in TAMs, future studies will be required to systematically dissect the relative contribution of these parallel pathways.

We observed that GC cells with elevated GULP1 expression served as a primary source of fatty acids within the TME. Metabolic heterogeneity in lipid utilization is increasingly recognized as a crucial candidate of tumor progression and immune modulation [39,40]. Cross talk between tumor and TAM lipid metabolism has been shown to drive T-cell exhaustion and to shape responsiveness to immunotherapy [41]. Among the regulators of lipogenesis, INSIG2 plays a pivotal role in controlling lipid synthesis. The INSIG1/2–SCAP–SREBP1 axis modulates fatty acid biosynthesis by retaining or releasing SCAP–SREBP1 complexes depending on intracellular sterol levels [28]. Previous studies have demonstrated that posttranslational phosphorylation of INSIG1/2 disrupts their association with SCAP, thereby promoting tumor progression [29,30]. Our findings uncovered a distinct regulatory mechanism in which GULP1, despite having no effect on INSIG expression or its phosphorylation, acetylation, or lactylation status (details provided in Table S3), markedly enhanced INSIG2 palmitoylation at cysteine 14. Protein palmitoylation—a reversible lipid modification—plays essential roles in diverse physiological processes including intracellular trafficking, signaling, and nutrient uptake. Functional assays revealed that palmitoylation of INSIG2 at Cys14 facilitated the dissociation of the SCAP–SREBP1 complex, leading to the activation of lipogenesis in GC cells. Although GULP1 itself lacks intrinsic enzymatic activity for lipid modification, LC–MS analysis identified that both GULP1 and INSIG2 interact with zDHHC8, a palmitoyltransferase located on the ER. Our data further established that INSIG2 serves as a substrate of zDHHC8-mediated palmitoylation. Notably, GULP1 functioned as an adaptor that strengthened the interaction between INSIG2 and zDHHC8, thereby promoting INSIG2 palmitoylation and stimulating de novo lipogenesis in GC cells. Moreover, the increased fatty acid synthesis driven by this pathway could, in turn, enhance the substrate availability for zDHHC8, forming a positive feedback loop that amplifies lipid metabolic reprogramming in the TME. In addition to the functional evidence presented in this study, the preferential involvement of zDHHC8 in the GULP1–INSIG2–SREBP1 axis may be partly explained by its subcellular localization. zDHHC8 is predominantly localized to the ER, where INSIG2 and SREBP1 are known to reside, potentially providing a spatial advantage that facilitates efficient interaction and palmitoylation of INSIG2 in response to GULP1. This spatial proximity may represent an important determinant of substrate selectivity among DHHC family members in this context. Moreover, emerging studies have highlighted a broader role for zDHHC8 in tumor biology. For example, zDHHC8 has been reported to promote the palmitoylation of GPX4 and SLC7A11, thereby enhancing tumor cell resistance to ferroptosis [42,43]. These findings, together with our data, suggest that zDHHC8 may function as a key regulator linking lipid modification processes to multiple tumor-promoting pathways, including metabolic remodeling and resistance to cell death. Nevertheless, given the potential redundancy within the DHHC family, we cannot exclude the contribution of other palmitoyltransferases under specific conditions, and further studies will be required to systematically define the substrate specificity and regulatory hierarchy of DHHC enzymes in the TME. Due to the lack of specific GULP1 inhibitors, we identified GA as a small molecule capable of binding to GULP1 to inhibit its function through virtual screening. GA has been previously recognized for its anti-tumor potential as an HMGB1 inhibitor [44,45], as well as a platform for delivery of plenty of anti-tumor drugs [46]. In our study, GA interfered with the association between GULP1 and INSIG2, thereby dampening zDHHC8-mediated INSIG2 palmitoylation, which restrained GC cell de novo fatty acid biosynthesis. Beyond its standalone anti-tumor efficacy, our study proved that GA markedly strengthened the therapeutic outcome of PD-1 blockade by inhibiting GULP1-mediated metabolic reprograming of TAMs. Combined treatment with GA and anti-PD-1 antibody produced pronounced tumor regression and reshaped the TME toward an immune-activated state, as evidenced by enhanced infiltration of GZMB^+^CD8^+^ T cells and MHC-II^+^ macrophages. Together, these results highlight GA as a promising pharmacologic agent that could be leveraged to improve the responsiveness of GC to ICB. Our findings raise the possibility that GULP1 may function as a broader metabolic and immune regulatory node across multiple tumor types. Previous studies have shown that GULP1 promotes tumor progression in hepatocellular carcinoma through the activation of β-catenin signaling and epithelial–mesenchymal transition [19], while pan-cancer analyses suggest that GULP1 is associated with poor prognosis, tumor progression, immune cell infiltration, and immunotherapy response, specifically pancreatic cancer, potentially through pathways such as HIPPO, mTOR, and RTK [20]. In addition, GULP1 has been implicated in the regulation of PPARG-mediated lipid metabolism and insulin-responsive glucose uptake in adipocyte models, further supporting its role in metabolic regulation [31]. In the present study, we extend these observations by demonstrating that in GC, GULP1 not only enhances tumor cell malignant behavior but also remodels the TME by promoting lipid-dependent metabolic reprogramming in TAMs, thereby impairing CD8^+^ T-cell function and contributing to resistance to anti-PD-1 therapy. Together, these findings suggest that GULP1 may integrate tumor signaling with metabolic and immune regulatory circuits within the TME. Although direct cross-tumor evidence remains limited, the convergence of oncogenic signaling, metabolic remodeling, and immune suppression observed here is consistent with the concept that key regulatory molecules can act as central nodes linking tumor progression with immune evasion. Interestingly, emerging evidence also suggests that the function of GULP1 may be context dependent across different tumor types. While our findings and previous studies in hepatocellular carcinoma and pancreatic cancer support a tumor-promoting role for GULP1, other reports indicate that GULP1 may exert tumor-suppressive effects in certain malignancies, such as ovarian and bladder cancers, where its down-regulation has been associated with enhanced proliferation and chemoresistance [18,47]. These seemingly divergent observations suggest that the biological function of GULP1 may be highly dependent on tumor context, cellular lineage, and the specific signaling or metabolic landscape. Such context-dependent roles are consistent with the notion that metabolic and immune regulatory molecules can exert distinct functions depending on the TME and cellular state.

Several limitations of this study should be acknowledged. Although we identified GULP1 as a potential prognostic and predictive biomarker in a prospective cohort, further validation in larger, multicenter studies will be essential to define a robust clinical cutoff and to evaluate its value for patient stratification in GC. Moreover, since protein palmitoylation has been shown to enhance fatty acid transport and secretion by stabilizing transporters such as CD36 and ABCA1, it is conceivable that GULP1 may also facilitate lipid export through stabilization of lipid transporters, thereby fostering a lipid-enriched TME [48]. Lastly, comprehensive pharmacokinetic and toxicological assessments are needed to fully characterize the specificity, safety, and clinical feasibility of GA prior to its potential therapeutic application.

## Conclusions

In summary, our findings reveal that GULP1 orchestrates metabolic reprogramming and immune suppression within the TME, thereby driving resistance to PD-1 blockade. Targeting the GULP1/zDHHC8/INSIG2/SREBP1 signaling axis with GA offers a promising strategy to restore anti-tumor immunity and enhance the efficacy of immunotherapy in GC.

## Ethical Approval

Written informed consent and clinical samples were obtained from all patients under the approval of the Ethics Committee of Zhongshan Hospital, Fudan University (No. B2024-261). The study was conducted in accordance with the Declaration of Helsinki and the International Conference on Harmonization Guidelines for Good Clinical Practice. All animal experiments were approved by the Ethics Committee of Zhongshan Hospital, Fudan University (No. Y2023-190) and were performed in accordance with institutional guidelines for the care and use of laboratory animals.

## Data Availability

Newly generated omics datasets have been deposited in the National Genomics Data Center (PRJCA067230 and PRJCA067228; https://ngdc.cncb.ac.cn/). All data supporting the findings of this study are available from the corresponding authors upon reasonable request.
