# Supplementary material for: GULP1 Fuels Resistance to PD-1 Blockade through Lipid-Mediated Metabolic Reprogramming of Tumor-Associated Macrophages in Gastric Cancer
Source: Cancer Commun (Lond). 2026 Oct 6;46:0054. doi: 10.34133/cancomm.0054 (PMC13639144; doi:10.34133/cancomm.0054)
Supplement: Supplementary file 1 [file cancomm.0054.f1.docx]

GULP1 Fuels Resistance to PD-1 Blockade through Lipid-Mediated Metabolic Reprogramming of Tumor-Associated Macrophages in Gastric Cancer

Zihao Zhang^1,2†^, Wenxin Xu^3†^, Kuan Yu^1,2†^, Anran Bao^1,2†^, Yifan Cao^1,2†^, Mincheng Yu^4^, Qiang Yu^3^, Yufei Zhao^3^, Liang Gong^1,2^, Peiyi Xie^3*^, Zhenbin Shen^1,2*^, Jing Qin^1,2*^

^1^Department of General Surgery, Zhongshan Hospital, Fudan University, Shanghai, P. R. China.

^2^Gastric Cancer Center, Zhongshan Hospital, Fudan University, Shanghai, P. R. China.

^3^Department of Hepatobiliary Surgery and Transplantation, Liver Cancer Institute, Key Laboratory of Carcinogenesis and Cancer Invasion of Ministry of Education, Zhongshan Hospital, Fudan University, Shanghai, P. R. China.

^4^Department of Colorectal Surgery, Zhongshan Hospital, Fudan University, Shanghai, P. R. China.

^†^These authors contributed equally.

*Corresponding Authors

**Jing Qin**, Department of General Surgery, Zhongshan Hospital, Fudan University, Shanghai, China; E-mail: qin.jing@zs-hospital.sh.cn; surgeonqin10458@163.com

**Zhenbin Shen**, Department of General Surgery, Zhongshan Hospital, Fudan University, Shanghai, China; E-mail: shen.zhenbin@zs-hospital.sh.cn

**Peiyi Xie**, Department of Hepatobiliary Surgery and Transplantation, Zhongshan Hospital, Fudan University, Shanghai, China; E-mail: [peiyi0505@163.com](mailto:peiyi0505@163.com)

Supplementary Figures

**Figure S1. Tumor intrinsic immunotherapy resistant gene screening and expression profile in GC patients.** (A) Differentially expressed genes (DEGs) between anti-PD-1 responders and non-responders depicted by volcano plot. (B) DEGs between anti-PD-1 sensitive and resistance mice depicted by volcano plot. (C) Average expression of known markers in different cell identities. (D) Heatmap of top five marker genes for each cell identities. (E) Immunotherapy resistance genes in non-responder patients and mice overlapping with tumor cell marker genes shown by Venn diagram. (F) List of 12 overlapping DEGs identified from the intersection of human non-responder samples and resistant mouse tumors. (G) Volcano plot showing differential gene expression between responders and non-responders, highlighting the relative upregulation of candidate genes, including GULP1. (H) mRNA level of *GULP1* in tumor and para-tumor specimens from 56 GC patients. (I) Representative image of immunoblotting analysis of GULP1 in tumor and para-tumor specimens from 56 GC patients. (J) Quantification of GULP1 of pre-treatment specimens from responders and non-responders in ZSGCI cohort. (K) ROC curve analysis assessing the predictive performance of GULP1 and established biomarkers for anti-PD-1 response.

Abbreviations: AADAC, Arylacetamide deacetylase; APOC3, Apolipoprotein C3; ERRFI1, ERBB receptor feedback inhibitor 1; FAM20A, FAM20A golgi associated secretory pathway pseudokinase; GPX3, Glutathione peroxidase 3; GULP1, GULP PTB domain containing engulfment adaptor 1; INSR, Insulin receptor; PD-1, Programmed cell death protein 1; PDK4, Pyruvate dehydrogenase kinase 4; RAMP1, Receptor activity modifying protein 1; RASD1, Ras related dexamethasone induced 1; SLCO2A1, Solute carrier organic anion transporter family member 2A1; UPK1B, Uroplakin 1B.

**Figure S2. GULP1 promotes GC progression *in vitro* and *in vivo*.** (A-B) GULP1 relative mRNA and protein level in four GC cell lines and a normal human gastric epithelial cell line GES-1. (C-D) GULP1 protein level in *GULP1* overexpression and knockdown GC cell lines compared with the control group. (E-F) *GULP1* overexpression on GC cell proliferation ability *in vitro* detected by EdU assay and colony formation assay. (G-H) *GULP1* knockdown on GC cell proliferation ability *in vitro* detected by EdU assay and colony formation assay. (I) *GULP1* overexpression on GC cell proliferation ability *in vivo* detected by mice subcutaneous tumor model (*n* = 6).

Abbreviations: EdU, 5-Ethynyl-2′-deoxyuridine

**Figure S3. Gulp1 mediated anti-PD-1 therapy resistance in a Cd8^+^ T cell dependent manner in immunocompetent mice.** (A-D) GULP1 relative mRNA and protein level in *Gulp1* overexpression and knockout YTN16 cell lines compared with the control group. (E) Representative images of the subcutaneous tumors in indicated groups at the endpoint (*n* = 6). (F, G) Volume and weight of subcutaneous tumors in indicated groups at the endpoint (*n* = 6). (H) Cd8^+^ T cell infiltration detected by mIF in indicated tumor groups. (I, J) Flow cytometric analysis and quantification of Gzmb^+^ Cd8^+^ cells in indicated tumor groups (*n* = 6). (K) Representative images of the subcutaneous tumors in indicated groups at the endpoint (*n* = 6). (L, M) Volume and weight of subcutaneous tumors in indicated groups at the endpoint (*n* = 6).

Abbreviations: DAPI, 4′,6-Diamidino-2-phenylindole; GFP, Green fluorescent protein; Gzmb, Granzyme B; IgG2a, Immunoglobulin G2a

**Figure S4. Expression of immune markers and corresponding statistical analysis according to CyTOF analysis.** (A) t-SNE plot of all Cd45^+^ cells colored by the expression levels of indicated markers. (B) Expression of important markers of all Cd45^+^ cells in *Gulp1* overexpression group compared with the control group (*n* = 3).

Abbreviations: Ly6g, Lymphocyte antigen 6 complex locus G; Tcrb, T-cell receptor beta; t-SNE, t-distributed stochastic neighbor embedding

**Figure S5. Specific markers expression in and T cell and macrophage population.** (A) T-SNE plot of markers expressed on T cell and macrophages. (B) Quantification of marker expression frequency in macrophage and T cell population.

Abbreviations: CCR2, C-C motif chemokine receptor 2; CX3CR1, C-X3-C motif chemokine receptor 1; FOXP3, Forkhead box P3; Ki-67, Marker of proliferation Ki-67; PD-L1, Programmed death-ligand 1; PD-L2, Programmed death-ligand 2; TIM-3, T-cell immunoglobulin and mucin-domain containing-3

**Figure S6. GULP1 expression negatively correlated with immune response in GC.** (A) Heatmap of immunological activity score calculated using ssGSEA in each tumor sample in TCGA-STAD cohort based on GULP1 high and low expression. (B) Box plot of the infiltration score of immune cell types calculated using ssGSEA in GULP1 high and low expression.

Abbreviations: ssGSEA, Single-sample gene set enrichment analysis

**Figure S7. GULP1 expression correlated with T cell and macrophage population in GC TME.** (A) UMAP and box plot of immune cells in GSE206785 based on GULP1 high and low expression. (B) Box plot of subclusters of macrophages in GSE206785 based on GULP1 high and low expression.

Abbreviations: TME, Tumor microenvironment; UMAP, Uniform manifold approximation and projection

**Figure S8. GULP1 recruited and reprogramed TAMs immunosuppressive phenotypes.** (A) Differential expression of immune suppression and activation related genes in each subclusters of macrophages. (B) Migration abilities of TAMs towards the supernatants from the indicated GC cell detected by Chemotaxis assays. (C) Immune suppression and activation related gene expression in BMDMs co-cultured with indicated YTN16 cells. (D-F) Flow cytometric analysis of Cd36^+^, Pd-l1^+^ and Mhc-ii^+^ of BMDMs co-cultured with indicated YTN16 cells. (G) CFSE assay detecting the inhibition of T-cell proliferation by BMDMs with different treatment as indicated. (H) Ifn-γ^+^ secretion by Cd8^+^ T cells in the assay in (H), measured by ELISA.

Abbreviations: BMDM, Bone marrow-derived macrophages; CFSE, Carboxyfluorescein succinimidyl ester; ELISA, Enzyme-Linked Immunosorbent Assay; H2-Ab1, histocompatibility 2, class II antigen A, beta 1; Mhc-ii, Major histocompatibility complex class II; Tgfb1, Transforming growth factor beta 1

**Figure S9. Validation of GULP1 expression and TME patterns in an independent scRNA-seq dataset.** (A) UMAP visualization of major cell populations in the GSE183904 GC dataset, including tumor epithelial cells, fibroblasts, endothelial cells, and immune cell subsets. (B) Feature plot showing GULP1 expression across all cell types. GULP1 expression is predominantly enriched in malignant epithelial cells and fibroblasts, with minimal expression observed in immune cell populations. (C) Quantification of the proportion of GULP1⁺ cells across different cell types, confirming the preferential expression of GULP1 in tumor and stromal compartments. (D) UMAP visualization of cell populations stratified by GULP1 expression status (GULP1-low vs. GULP1-high). (E) Comparison of cell-type composition between GULP1-low and GULP1-high tumors, showing increased macrophage abundance and reduced CD8⁺ T-cell proportion in GULP1-high tumors. (F) UMAP visualization of macrophage subclusters under GULP1-low and GULP1-high conditions. (G) Relative proportions of macrophage subclusters, indicating enrichment of macrophage cluster 1 in GULP1-high tumors. (H) Dot plot showing pathway enrichment across macrophage subclusters, highlighting increased fatty acid metabolism-related pathways in cluster 1. (I) Heatmap of lipid metabolism-related gene expression across macrophage subclusters, demonstrating elevated expression of FAO- and lipid handling-associated genes in cluster 1.

Abbreviations: FAO, Fatty acid oxidation; GSE, Gene expression omnibus series

**Figure S10. Identification of lipid-associated macrophage subsets in gastric cancer.** (A-C) UMAP feature plots showing the expression of CD36 (A), FABP5 (B), and TREM2 (C) across macrophage clusters in the single-cell RNA-seq dataset.

Abbreviations: FABP5, Fatty acid-binding protein 5; TREM2, Triggering Receptor Expressed on Myeloid cells 2

**Figure S11. GULP1 expression negatively correlated with CD8^+^ T cell infiltration in GC.** (A) mIF analysis of Cd8^+^ and F4/80^+^ immune cells in subcutaneous tumor tissues. Cd36^+^ F4/80^+^ cells are indicated by arrows. (B) Quantification for the number of Cd36^+^ F4/80^+^ cells and Cd8^+^ T cells per field (Right panel). (C) Correlation analysis between the Cd36^+^ F4/80^+^ cells and Cd8^+^ T cells. (D) Quantification of Cd36⁻ macrophages in vector and *Gulp1*-overexpressing tumors. (E) Correlation analysis between Cd36⁻ macrophages and Cd8⁺ T-cell abundance, showing a weak positive association. (F-L) Correlation analyses between CD8⁺ T-cell proportion and individual macrophage subclusters identified from GSE183904.

Abbreviations: Multiplex immunofluorescence

**Figure S12. GULP1 enhanced lipid metabolism signaling in TAMs.** (A) Lipid metabolism related gene expression in BMDMs co-cultured with indicated YTN16 cells. (B) Basal respiration of BMDMs co-cultured with indicated YTN16 cells according to OCR.

Abbreviations: Cpt1a, Carnitine palmitoyltransferase 1A;, Dgat1, Diacylglycerol O-acyltransferase 1; Fabp4, Fatty acid-binding protein 4; OCR, Oxygen consumption rate; Plin2, Perilipin 2; Pparg, Peroxisome proliferator-activated receptor gamma

**Figure S13. *Cd36* myeloid knockout reshaped the GC TME.** (A) mIF analysis of Cd8^+^ and F4/80^+^ immune cells in subcutaneous tumor tissues. Representative cells are indicated by arrows, including Mhc-ii^+^ F4/80^+^ cells and Gzmb^+^ Cd8^+^ T cells. (B) Number of Mhc-ii^+^ F4/80^+^ cells and Gzmb^+^ Cd8^+^ T cells per field in indicated tumor groups.

Abbreviations: Cre, Cyclization recombinase; Lyz2, Lysozyme 2;

**Figure S14. Cell-cell crosstalk in TME.** (A) Cell-cell communications between the identified cell types. (B) The outgoing and incoming signaling pathways of each cell subcluster.

Abbreviations: ANGPTL, Angiopoietin-like; ANNEXIN, Annexin; APRIL, A proliferation-inducing ligand; BAFF, B-cell activating factor; BAG, Bcl-2-associated athanogene; BMP, Bone morphogenetic protein; CALCR, Calcitonin receptor; CCL, C-C motif chemokine ligand; CSF, Colony-stimulating factor; CXCL, C-X-C motif chemokine ligand; EDN, Endothelin; EGF, Epidermal growth factor; EPHB, Ephrin type-B receptor; FASLG, Fas ligand; FGF, Fibroblast growth factor; GAS, Growth arrest-specific; GDF, Growth differentiation factor; GH, Growth hormone; GRN, Granulin; HGF, Hepatocyte growth factor; IGF, Insulin-like growth factor; IL, Interleukin; KIT, KIT proto-oncogene receptor tyrosine kinase; LIFR, Leukemia inhibitory factor receptor; MK, Midkine; MIF, Macrophage migration inhibitory factor; OSM, Oncostatin M; PARs, Protease-activated receptors; PDGF, Platelet-derived growth factor; PERIOSTIN, Periostin; PROS, Protein S; PTN, Pleiotrophin; RESISTIN, Resistin; SEMA3, Semaphorin 3; SPP1, Secreted phosphoprotein 1; TGFβ, Transforming growth factor beta; TNF, Tumor necrosis factor; TWEAK, TNF-related weak inducer of apoptosis; VEGF, Vascular endothelial growth factor; VISFATIN, Visfatin

**Figure S15. *Fasn* knockdown rescued Gulp1 medicated immune suppression of TAMs.** (A-C) Flow cytometric analysis of Cd36^+^, Pd-l1^+^ and Mhc-ii^+^ of BMDMs co-cultured with indicated YTN16 cells. (D) CFSE assay detecting the inhibition of T-cell proliferation by BMDMs with different treatment as indicated. (E) Ifn-γ^+^ secretion by Cd8^+^ T cells in the assay in (D), measured by ELISA.

Abbreviations: Fasn, Fatty acid synthase; TAMs, Tumor-associated macrophages

**Figure S16. *Fasn* knockdown rescued Gulp1 mediated tumor progression and TAMs reprogramming.** (A-B) Volume and weight of subcutaneous tumors in indicated groups (*n* = 6). (C) mIF analysis of Cd36^+^ F4/80^+^ immune cells in subcutaneous tumor tissues. Representative cells are indicated by arrows.

Abbreviations: Fasn, Fatty acid synthase; mIF, Multiplex immunofluorescence; TAMs, Tumor-associated macrophages

**Figure S17. GULP1 promoted the SCAP translocation from endoplasmic reticulum to Golgi apparatus.** (A) Immunofluorescence analyses of SCAP and calnexin in indicated GC cells. (B) Immunofluorescence analyses of SCAP and golgin97 in indicated GC cells. (C) Immunofluorescence analyses of SREBP1 in indicated GC cells

Abbreviations: SCAP, SREBP cleavage-activating protein; SREBP1, Sterol regulatory element-binding protein 1

**Figure S18. Immunoblotting analyzes of anti-INSIG2 immunoprecipitates derived from indicated GC cells.**

Abbreviations: Ace-lys, Acetyl-lysine; INSIG2, Insulin-induced gene 2; IP, Immunoprecipitation; Kla, Lysine lactylation; pan-Ace-lys, Pan-acetyl-lysine; pan-Kla, Pan-lysine lactylation; pan-pS, Pan-phosphoserine; WB, Western blotting

**Figure S19. Schematic illustration of INSIG2 mutants.**

Abbreviations: INSIG2, Insulin-induced gene 2

**Figure S20. INSIG2 palmitoylation partially reversed the SREBP1 induced lipogenesis.** (A) Western blotting analysis of SREBP1 and lipogenesis proteins in indicated GC cells. (B) mIF analyses of SREBP1 in indicated GC cells. (C) Representative images of BODIPY 493/503 staining of BMDMs co-cultured with indicated YTN-16 cells. (D) OCR of BMDMs co-cultured with indicated YTN-16 cells and quantification of basal respiration in indicated TAMs. (E) Flow cytometric analysis of Cd36^+^ and Pd-l1^+^ of BMDMs co-cultured with indicated YTN-16 cells. (G) CFSE assay detecting the inhibition of T-cell proliferation by BMDMs with different treatment as indicated. (H) Ifn-γ^+^ secretion by Cd8^+^ T cells in indicated groups measured by ELISA.

Abbreviations: ACC1, Acetyl-CoA carboxylase 1; SCD1, Stearoyl-CoA desaturase 1

**Figure S21. zDHHC8 regulated the SREBP1 induced lipogenesis.** (A) Western blotting analysis of SREBP1 and lipogenesis proteins in indicated GC cells. (B) mIF analyses of SREBP1 in indicated GC cells. (C) Representative images of BODIPY 493/503 staining of BMDMs co-cultured with indicated YTN-16 cells. (D) OCR of BMDMs co-cultured with indicated YTN-16 cells and quantification of basal respiration in indicated TAMs. (E) Flow cytometric analysis of Cd36^+^ and Pd-l1^+^ of BMDMs co-cultured with indicated YTN-16 cells. (G) CFSE assay detecting the inhibition of T-cell proliferation by BMDMs with different treatment as indicated. (H) Ifn-γ^+^ secretion by Cd8^+^ T cells in indicated groups measured by ELISA.

Abbreviations: zDHHC8, Zinc finger DHHC-type palmitoyltransferase 8

**Figure S22. GULP1 inhibitor screening.** (A) Flow chart depicting the screening strategy for small-molecule compounds targeting GULP1. (B) Schematic diagram of the predicted docking structure of GA and GULP1.

Abbreviations: GA, Glycyrrhizic acid; HTS, High-throughput screening

**Figure S23. GA inhibited the SCAP translocation from endoplasmic reticulum to Golgi apparatus.** (A) Immunofluorescence analyses of SCAP and calnexin in indicated GC cells. (B) Immunofluorescence analyses of SCAP and golgin97 in indicated GC cells. (C) Immunofluorescence analyses of SREBP1 in indicated GC cells.

Abbreviations: GA, Glycyrrhizic acid; SCAP, SREBP cleavage-activating protein; SREBP1, Sterol regulatory element-binding protein 1

**Figure S24. GA reversed GULP1 mediated immune suppression of TAMs by inhibiting GC cell *de novo* fatty acid biosynthesis *in vitro*.** (A) Representative images of BODIPY 493/503 staining of indicated YTN16 cells. (B) Metabolomic profiling of long-chain UFAs in indicated YTN16 cells. (C) BODIPY-C16 levels in the indicated YTN16 cells and in BMDMs co-cultured with the respective YTN16 cells. (D) Representative images of BODIPY 493/503 staining of BMDMs co-cultured with indicated YTN16 cells. (E) Basal respiration of BMDMs co-cultured with indicated YTN16 cells according to OCR. (F) Immune suppression and activation related gene expression of BMDMs co-cultured with indicated YTN16 cells.

Abbreviations: OA, Oleic acid; PA, Palmitic acid; SA, Stearic acid

**Figure S25. GA treatment reversed Gulp1 mediated hindered Cd8^+^ T cell cytotoxic function by inhibiting Cd36^+^ TAMs accumulation *in vivo*.** (A, B) Volume and weight of subcutaneous tumors in indicated groups (*n* = 6). (C) mIF analysis of Cd36^+^ F4/80^+^ immune cells in subcutaneous tumor tissues. Representative cells are indicated by arrows. (D) Flow cytometric analysis and quantification of Cd36^+^ F4/80^+^ TAMs and Gzmb^+^ Cd8^+^ cells in indicated tumor groups (*n* = 6).

Abbreviations: GA, Glycyrrhizic acid; GULP1, GULP PTB domain containing engulfment adaptor 1; Gzmb, Granzyme B

**Figure S26. Targeting GULP1 in PDOs reversed GULP1 high expression-induced anti-PD-1 resistance and enhanced anti-PD-1 therapy efficiency.** (A) PDOs were treated by anti-PD-1 antibody (250 μg/mL) and/or GA (50 μmol/L). Representative images in PDO-01 and 05 in indicated groups were presented. (B) Quantification of PDOs from GULP1 high and low expression patients treated by indicated therapy.

Abbreviations: Combo, Combination treatment; PDOs, Patient-derived organoids

Supplementary Tables

**Table S1. Clinicopathological Characteristics of ZSGCI Patients by therapy responsiveness**

| **Characteristics** | **Responder** | | **Non-Responder** | | ***P* value** | |
| --- | --- | --- | --- | --- | --- | --- |
| **All patients** | 48 | 34 | |  | |  |
| **Age (years)^a^** | | | | | 0.469 | |
| Mean (SD) | 63.58 (10.81) | | 61.71 (11.89) | |  | |
| **Sex** | | | | | 0.978 | |
| Female | 10 | | 7 | |  | |
| Male | 38 | | 27 | |  | |
| **Grade** | | | | | 0.681 | |
| G1 | 6 | | 5 | |  | |
| G2 | 12 | | 11 | |  | |
| G3 | 30 | | 18 | |  |  |
| **Lauren classification** | | | | | 0.099 | |
| Diffuse | 30 | | 15 | |  | |
| Intestinal | 18 | | 19 | |  | |
| **Localization** | | | | | 0.835 | |
| Proximal | 16 | | 10 | |  | |
| Middle | 18 | | 12 | |  |  |
| Distal | 14 | | 12 | |  |  |
| **TNM stage** | | | | | 0.752 | |
| I | 12 | | 7 | |  | |
| II | 16 | | 10 | |  |  |
| III | 20 | | 17 | |  |  |
| **GULP1 Level** | | | | | **0.035** | |
| High | 17 | | 20 | |  | |
| Low | 31 | | 14 | |  |  |

Abbreviations: GULP1, GULP PTB domain containing engulfment adaptor 1; SD, Standard deviation; TNM, Tumor-node-metastasis

**Table S2. Clinicopathological Characteristics of ZSGC Patients by GULP1 expression**

| **Factor** | **GULP1-low** | **GULP1-high** | ***P* value** |  |
| --- | --- | --- | --- | --- |
| **All patients** | 67 | 68 |  |  |
| **Age(years)^a^** |  |  | 0.057 |  |
| Mean (SD) | 56.88 (13.10) | 60.79 (10.50) |  |  |
| **Sex** |  |  | 0.807 |  |
| Female | 23 | 22 |  |  |
| Male | 44 | 46 |  |  |
| **Grade** |  |  | 0.888 |  |
| G1 | 8 | 10 |  |  |
| G2 | 24 | 23 |  |  |
| G3 | 35 | 35 |  |  |
| **Lauren classification** |  |  | 0.431 |  |
| Diffuse | 39 | 35 |  |  |
| Intestinal | 28 | 33 |  |  |
| **T classification** |  |  | **0.009** |  |
| T1 | 15 | 9 |  |  |
| T2 | 18 | 8 |  |  |
| T3 | 21 | 22 |  |  |
| T4 | 13 | 29 |  |  |
| **N classification** |  |  | 0.080 |  |
| N0 | 8 | 14 |  |  |
| N1 | 23 | 11 |  |  |
| N2 | 18 | 19 |  |  |
| N3 | 18 | 24 |  |  |
| **TNM stage** |  |  | **0.002** |  |
| I | 18 | 9 |  |  |
| II | 24 | 13 |  |  |
| III | 25 | 46 |  |  |

Abbreviations: GULP1, GULP PTB domain containing engulfment adaptor 1; SD, Standard deviation; TNM, Tumor-node-metastasis

**Table S3. Sequences of the primers used in the study**

| PCR primer for genotyping | | | |
| --- | --- | --- | --- |
| Genes | | **Sequences (5’-3’)** | |
| *Lyz2*- primer1 | | 5′ CCCAGAAATGCCAGATTACG 3′ | |
| *Lyz2*- primer2 | | 5′ CTTGGGCTGCCAGAATTTCTC 3′ | |
| *Lyz2*- primer3 | | 5′ TTACAGTCGGCCAGGCTGAC 3′ | |
| *Cd36* forward | | 5′ GCCTGCATGGAGTGCATAGT 3′ | |
| *Cd36* reverse | | 5′ CATGCCTGAAACTTTCCCCA 3′ | |
| sgRNA and shRNA target sites | | | |
| Species | **Gene** | **Sequences (5’-3’)** | **PAM (sg)** |
| Mouse | *Gulp1*-1 | GCAATACCAGTATGGAGCCA | TGG |
| Mouse | *Gulp1*-2 | ACTTATGCATTGGCATGTCC | AGG |
| Human | sh*GULP1* | AGTTTCTTGGCAGTACCGA | |
| Human | sh*FASN* | CAGATGATGGAGGAGTTCA | |
| PCR primer pairs | | | |
| Human *GULP1* | forward | AGAGCAATGAAAGGGATGTA | |
| Human *GULP1* | reverse | TCAGAGATGTTGATGCTTTCA | |
| Mouse *Cd36* | forward | GGACATTGAGATTCTTTTCCTCTG | |
| Mouse *Cd36* | reverse | GCAAAGGCATTGGCTGGAAGAAC | |
| Mouse *Pparg* | forward | GTACTGTCGGTTTCAGAAGTGCC | |
| Mouse *Pparg* | reverse | ATCTCCGCCAACAGCTTCTCCT | |
| Mouse *Dgat1* | forward | GGTTCCGTGTTTGCTCTGGCAT | |
| Mouse *Dgat1* | reverse | CCACTGACCTTCTTCCCTGTAG | |
| Mouse *Plin2* | forward | CTGGAGGAAGGTGACTACAGT | |
| Mouse *Plin2* | reverse | TCAGATGTCAGTCACCATCAT | |
| Mouse *Fabp4* | forward | GATGCCTTTGTGGGAACCTGG | |
| Mouse *Fabp4* | reverse | GCTGCATTGGGGTTCAACACT | |
| Mouse *Cpt1a* | forward | GGCATAAACGCAGAGCATTCCTG | |
| Mouse *Cpt1a* | reverse | CAGTGTCCATCCTCTGAGTAGC | |
| Mouse *Cd274* | forward | GATCAGACTCCAACTCTACCG | |
| Mouse *Cd274* | reverse | TGATCTGAAGGGCAGCATTTC | |
| Mouse *Tgfb1* | forward | TGATACGCCTGAGTGGCTGTCT | |
| Mouse *Tgfb1* | reverse | CACAAGAGCAGTGAGCGCTGAA | |
| Mouse *Il10* | forward | CGGGAAGACAATAACTGCACCC | |
| Mouse *Il10* | reverse | CGGTTAGCAGTATGTTGTCCAGC | |
| Mouse *H2-Ab1* | forward | CCCAAGACATTCCGATTTCGT | |
| Mouse *H2-Ab1* | reverse | GGCGTTCCTTCATAAACCTCT | |
| Mouse *Cd86* | forward | CTTCACAGCCACGGATCAG | |
| Mouse *Cd86* | reverse | GCTGATCCAAGGATCAACTGA | |
| Mouse *Gapdh* | forward | TGCACCACCAACTGCTTAG | |
| Mouse *Gapdh* | reverse | GGATGCAGGGATGATGTTC | |

Abbreviations: Cpt1a, Carnitine palmitoyltransferase 1A; Dgat1, Diacylglycerol O-acyltransferase 1; Fabp4, Fatty acid-binding protein 4; FASN, Fatty acid synthase; Gapdh, Glyceraldehyde-3-phosphate dehydrogenase; GULP1, GULP PTB domain containing engulfment adaptor 1; H2-Ab1, Histocompatibility 2, class II antigen A, beta 1; Il10, Interleukin 10; Lyz2, Lysozyme 2; Plin2, Perilipin 2; Pparg, Peroxisome proliferator-activated receptor gamma; Tgfb1, Transforming growth factor beta 1

**Table S4. Details of 42 antibodies applied in CyTOF**

| Label | Marker | Clone | Company | Cat**alog number** | Dilution |
| --- | --- | --- | --- | --- | --- |
| 89Y | CD45 | 30-F11 | Biolegend | 103102 | 1:400 |
| 115ln | CD3ε | 145-2C11 | Biolegend | 100302 | 1:100 |
| 139La | Ki-67 | SolA15 | Biolegend | 14-5698-82 | 1:400 |
| 141Pr | CD36 | HM | Biolegend | 559921 | 1:100 |
| 142Nd | MHC II (I-A/I-E) | M5/114.15.2 | Biolegend | 102602 | 1:400 |
| 143Nd | CD172a (SIRPα) | P84 | Biolegend | 144002 | 1:50 |
| 144Nd | CX3CR1 | SA011F11 | Biolegend | 149002 | 1:400 |
| 145Nd | CD38 | 90 | Biolegend | 102702 | 1:400 |
| 146Nd | CD206 (MMR) | C068C2 | Biolegend | 141702 | 1:50 |
| 147Sm | CD73 | TY/11.8 | Biolegend | 127202 | 1:200 |
| 148Nd | CD169 (Siglec-1) | 3D6.112 | Biolegend | 142402 | 1:50 |
| 149Sm | CD19 | 6D5 | Biolegend | 115502 | 1:800 |
| 150Nd | Ly-6C | HK1.4 | Biolegend | 128002 | 1:400 |
| 151Eu | CD44 | IM7 | Biolegend | 103002 | 1:200 |
| 152Sm | CD11c | N418 | Biolegend | 117302 | 1:100 |
| 153Eu | CD274 (PD-L1) | 10F.9G2 | Biolegend | 124302 | 1:200 |
| 154Sm | CD62L | MEL-14 | Biolegend | 104402 | 1:200 |
| 155Gd | CD103 | 2E7 | Biolegend | 121402 | 1:200 |
| 156Gd | FOXP3 | FJK-16s | eBioscience | 14-5773-82 | 1:50 |
| 157Gd | CD83 | Michel-19 | Biolegend | 121502 | 1:100 |
| 158Gd | CD86 | GL-1 | Biolegend | 105002 | 1:400 |
| 159Tb | CD279 (PD-1) | 29F.1A12 | Biolegend | 135202 | 1:200 |
| 160Gd | CD115 (CSF-1R) | AFS98 | Biolegend | 135502 | 1:200 |
| 161Dy | CD14 | Sa14-2 | Biolegend | 123302 | 1:100 |
| 162Dy | CD25 (IL-2Rα) | 3C7 | Biolegend | 101902 | 1:200 |
| 163Dy | Tim-4 | RMT4-54 | Biolegend | 130002 | 1:400 |
| 164Dy | CD192 (CCR2) | 475301 | RD | MAB55381-100 | 1:200 |
| 165Ho | CD64 (FcγRI) | X54-5/7.1 | Biolegend | 139302 | 1:50 |
| 166Er | Ly-6G | 1A8 | Biolegend | 127602 | 1:200 |
| 167Er | F4/80 | Cl:A3-1 | Biorad | MCA497G | 1:100 |
| 168Er | CD49b (pan-NK cells) | DX5 | Biolegend | 108902 | 1:400 |
| 169Tm | CD273 (PD-L2) | TY25 | Biolegend | 107202 | 1:50 |
| 170Er | iNOS | CXNFT | eBioscience | 14-5920-82 | 1:50 |
| 171Yb | CD69 | H1.2F3 | Biolegend | 104502 | 1:100 |
| 172Yb | CD182 (CXCR2) | SA044G4 | Biolegend | 149302 | 1:100 |
| 173Yb | Granzyme B Recombinant | QA16A02 | Biolegend | 372202 | 1:50 |
| 174Yb | CD27 | LG.3A10 | Biolegend | 124202 | 1:50 |
| 175Lu | TCR β chain | H57-597 | Biolegend | 109202 | 1:100 |
| 176Yb | CD366 (Tim-3) | RMT3-23 | Biolegend | 119702 | 1:200 |
| 197Au | CD4 | RM4-5 | Biolegend | 100576 | 1:800 |
| 198Pt | CD8a | 53-6.7 | Biolegend | 100746 | 1:200 |
| 209Bi | CD11b | M1/70 | Biolegend | 101202 | 1:800 |

Abbreviations: CCR2, C-C motif chemokine receptor 2; CSF-1R, Colony-stimulating factor 1 receptor; CX3CR1, C-X3-C motif chemokine receptor 1; CXCR2, C-X-C motif chemokine receptor 2; FOXP3, Forkhead box P3; iNOS, Inducible nitric oxide synthase; IL-2Rα, Interleukin-2 receptor alpha; MHC II, Major histocompatibility complex class II; MMR, Mannose receptor; PD-1, Programmed cell death protein 1; PD-L1, Programmed death-ligand 1; PD-L2, Programmed death-ligand 2; SIRPα, Signal regulatory protein alpha; TCR, T-cell receptor; TIM-3, T-cell immunoglobulin and mucin-domain containing-3; TIM-4, T-cell immunoglobulin and mucin-domain containing-4

**Table S5. Primary antibodies used in present study**

| Antibodies | Concentration | Application | Identifier | Company |
| --- | --- | --- | --- | --- |
| GULP1 | 1:1000 | Western blot | Cat# 19902-1-AP | Proteintech |
|  | 1:100 | IP |  |  |
|  | 1:400 | IF |  |  |
|  | 1:600 | IHC |  |  |
| CD36 | 1:100 | IF | Cat# 28109 | Cell Signaling Technology |
| INSIG2 | 1:1000 | Western blot | Cat# 24766-1-AP | Proteintech |
|  | 1:400 | IF |  |  |
|  | 1:100 | IP |  |  |
| SCAP | 1:500 | Western blot | Cat# ab190103 | Abcam |
|  | 1:100 | IF |  |  |
| Calnexin | 1:100 | IF | Cat# 66903-1-Ig | Proteintech |
| Golgin97 | 1:100 | IF | Cat# 13192 | Cell Signaling Technology |
| SREBP1 | 1:1000 | Western blot | Cat# 14088-1-AP | Proteintech |
|  | 1:100 | IF |  |  |
| Anti-Phospho-Tyrosine | 1:1000 | Western blot | Cat# 8954 | Cell Signaling Technology |
| Anti-Acetylated-Lysine | 1:1000 | Western blot | Cat# 9441 | Cell Signaling Technology |
| Anti-L-Lactyl Lysine | 1:1000 | Western blot | Cat# PTM-1401RM | PTM BIO |
| Anti-Flag-Tag | 1:1000 | Western blot | Cat# 66008-4-Ig | Proteintech |
|  | 1:100 | IP |  |  |
| Anti-HA-Tag | 1:1000 | Western blot | Cat# 51064-2-AP | Proteintech |
|  | 1:100 | IP |  |  |
| CD8a | 1:100 | IHC | Cat# ab237709 | Abcam |
|  | 1:100 | IF |  |  |
| GZMB | 1:100 | IF | Cat# ab317459 | Abcam |
| F4/80 | 1:100 | IF | Cat# ab6640 | Abcam |
| MHC-II | 1:100 | IF | Cat# ab180779 | Abcam |
| α-Tubulin | 1:1000 | Western blot | Cat# 2125 | Cell Signaling Technology |
| BUV395 Rat Anti-Mouse CD45 | 5µl/million cells | Flow cytometry | Cat# 565967 | BD Bioscience |
| PE/Cy7 anti-mouse CD3 | 5µl/million cells | Flow cytometry | Cat# 100219 | Biolegend |
| FITC anti-mouse CD8a | 5µl/million cells | Flow cytometry | Cat# 162313 | Biolegend |
| Brilliant Violet 605™ anti-mouse CD4 Antibody | 5µl/million cells | Flow cytometry | Cat# 100451 | Biolegend |
| Brilliant Violet 421™ anti-human/mouse Granzyme B | 5µl/million cells | Flow cytometry | Cat# 515409 | Biolegend |
| Brilliant Violet 510™ anti-mouse IFN-γ Antibody | 5µl/million cells | Flow cytometry | Cat# 505841 | Biolegend |
| FITC anti-mouse/human CD11b Antibody | 5µl/million cells | Flow cytometry | Cat# 101205 | Biolegend |
| PerCP/Cyanine5.5 anti-mouse F4/80 Antibody | 5µl/million cells | Flow cytometry | Cat# 123127 | Biolegend |
| Brilliant Violet 605™ anti-mouse Ly-6G Antibody | 5µl/million cells | Flow cytometry | Cat# 127639 | Biolegend |
| Brilliant Violet 650™ anti-mouse I-A/I-E Antibody | 5µl/million cells | Flow cytometry | Cat# 107641 | Biolegend |
| PE anti-mouse CD36 Antibody | 5µl/million cells | Flow cytometry | Cat# 102605 | Biolegend |
| Brilliant Violet 421™ anti-mouse CD274 (B7-H1, PD-L1) Antibody | 5µl/million cells | Flow cytometry | Cat# 124315 | Biolegend |
| eBioscience™ Fixable Viability Dye eFluor™ 780 | 5µl/million cells | Flow cytometry | Cat# 2633407 | Invitrogen |

Abbreviations: B7-H1, B7 homolog 1; BUV, Brilliant ultraviolet; BV, Brilliant Violet; Cy7, Cyanine 7; FITC, Fluorescein isothiocyanate; GULP1, GULP PTB domain containing engulfment adaptor 1; GZMB, Granzyme B; HA, Hemagglutinin; IFN-γ, Interferon gamma; INSIG2, Insulin-induced gene 2; MHC-II, Major histocompatibility complex class II; PD-L1, Programmed death-ligand 1; PE, Phycoerythrin; PerCP, Peridinin-chlorophyll-protein complex; SCAP, SREBP cleavage-activating protein; SREBP1, Sterol regulatory element-binding protein 1
